# Longitudinal *in vivo* imaging of dynamic immune–vascular responses to meningeal cerebrovascular injury

**DOI:** 10.1186/s12974-026-03767-6

**Published:** 2026-03-17

**Authors:** Aurora Kraus, Jean S. Prosper-Santiago, Aleksandra Potapova, John Prevedel, Daniel Castranova, Brant M. Weinstein

**Affiliations:** 1 Division of Developmental Biology, Eunice Kennedy Shriver National Institute of Child Health and Human Development, NIH, Bethesda, MD USA; 2 Section on Vertebrate Organogenesis, Building 6B, Room 4B413 6 Center Drive, Bethesda, MD 20892 USA

**Keywords:** Meningeal vasculature, Cerebrovascular injury, Inflammation, Live imaging, Zebrafish

## Abstract

Meningeal cerebrovascular injuries (mCVI) trigger prolonged bleeding and inflammation that contribute to long-term neurological dysfunction, yet the immune–vascular dynamics governing meningeal repair remain poorly understood. Existing animal models limit direct observation of these processes due to invasive procedures and restricted optical access through opaque skulls. With a thin, translucent skull, the zebrafish provides an idea model for longitudinal imaging of mCVI sequelae in intact, unmanipulated animals. We have established an adult zebrafish model to investigate mCVI using precisely calibrated sonication to rupture meningeal blood vessels without breaching the skull or damaging the underlying brain. Using longitudinal live imaging of intubated adult zebrafish, we directly visualize vascular disruption, blood flow dynamics and preservation, angiogenic regrowth and pruning, and immune cell population dynamics over time in the same animal with unprecedented resolution. Together, these studies unveil the meninges as a dynamic immune–vascular niche and demonstrate that our newly developed zebrafish mCVI model provides a powerful platform for longitudinal live imaging, opening the door to new insights into chronic neuroinflammatory disease at CNS borders.

**Supplementary Information:**

The online version contains supplementary material available at 10.1186/s12974-026-03767-6.

## Introduction

Although few immune cells infiltrate a healthy brain, it is surrounded by the meninges, a set of immune cell-rich tissue layers that protect vertebrate brains [1–7]. Zebrafish, mouse, and human meninges have remarkable anatomical conservation in the construction of the dura mater and leptomeningeal layers, immune cell composition of each layer, and meningeal vascular networks [7–16]. Head trauma often damages meningeal blood vessels, causing meningeal hematomas, inflammation and meningeal cerebrovascular injuries (mCVI) [17]. Head traumas can cause lasting disease pathologies with limited treatment options [18–20]. Acute inflammation facilitates debris clean-up and triggers regeneration, but persistent inflammation correlates with poor long term clinical outcomes [21–26]. Patients with prolonged vascular leakage in the meninges are more likely to have poor clinical outcomes [27, 28], and the feedback between meningeal vascular leakage and inflammation is thought to be important in long-term pathology post-mCVI.

A variety of animal models are available to study post-head trauma pathologies, but most of these models focus on neuronal histology, inflammation, and blood brain barrier (BBB) disruption within the brain itself without assessing trauma specific to the surrounding meninges (reviewed in [29–35]). The recent rediscovery of meningeal lymphatic vessels in the dura of vertebrates [14–16], has also piqued interest in exploring meningeal functions beyond their traditional role as a barrier for maintaining brain homeostasis [36, 37]. Many types of head injuries can rupture blood vessels in the superficial and highly vascularized meninges, and a few models have been developed with the aim of replicating meningeal vascular pathologies observed in humans. A murine model of subarachnoid hemorrhage involving autologous blood transfer to the magna cisterna to study vascular function after meningeal hematoma revealed that dural lymphatic vessels play a role in debris clearance and modulate neuroinflammation [38–40]. A murine compression injury model was used for imaging myeloid cell accumulation around meningeal vascular wounds and their role in vascular repair [23, 41]. A transcranial ultrasound CVI model was also used in mice to examine how the inflammatory state of the meninges impacts vascular repair [42, 43]. However, use of each of these models for observation of the meninges involves surgeries and punctures that themselves result in coarse meningeal vascular injuries that cause neuroinflammation and obscure the native response. Together, these limitations have restricted our ability to directly observe how immune cells interact with meningeal blood vessels over time following injury, particularly during the transition from acute inflammation to vascular repair. An ideal model would permit non-invasive, longitudinal imaging of immune and vascular dynamics within intact meninges, while minimizing confounding injury-induced inflammation.

The structural and functional similarities between the neural, vascular, and immune systems of zebrafish and mammals make the fish well-suited for investigating vascular and immune responses to mCVI [44–47]. The availability of innumerable immune cell type-specific transgenic reporter lines has made it possible to carry out live imaging of immune cell dynamics, including new insights into neutrophil migration after injury in larval zebrafish [48, 49]. Longitudinal high-resolution time-lapse imaging of immune cells is possible in adult zebrafish using a non-surgical intubation technique [50, 51]. Using this technique, immune cells and vessels can be clearly imaged through the thin, translucent skull of the adult zebrafish, making it possible to use intubation to carry out high-resolution non-invasive live time-lapse and/or longitudinal imaging. As noted above, zebrafish have mammalian-like meninges including both dura mater and leptomeningeal layers, which have been shown to contain cells similar to those found in mammals [8]. As a longstanding model for development and neuroscience, zebrafish also offer a wide array of genetic tools and cell-specific reporters, as well as validated behavioral tests that can be used to relate injuries to organism-wide consequences and outcomes relevant to humans [52]. Indeed, zebrafish have long been used as models for traumatic brain and spinal cord injuries, but as in mouse models, most zebrafish models developed to date either penetrate the skull or are technically prohibitive and focus on damage to nervous tissues [53–56].

Here, we establish a non-invasive zebrafish model of meningeal cerebrovascular injury that enables longitudinal in vivo imaging of immune–vascular dynamics during inflammation and repair. This new model generates a focal, sterile meningeal cerebrovascular injury that produces vascular disruption and hemorrhage while sparing the brain parenchyma, with the goal of modeling clinically relevant minor head trauma involving meningeal vessels. To accomplish this, we adapted and calibrated a dental sonication tool to induce focal mCVI. Behavioral analysis after injury shows these injuries elicit only minor aberrant anxiety responses, reflecting the lack of brain damage we observe. We establish that transgenic vascular and immune reporter lines enable high-resolution, longitudinal in vivo imaging of dynamic inflammatory responses and vascular remodeling following meningeal cerebrovascular injury.

## Results

### An adult zebrafish model of sterile dural meningeal cerebrovascular injury

**Fig. 1:**
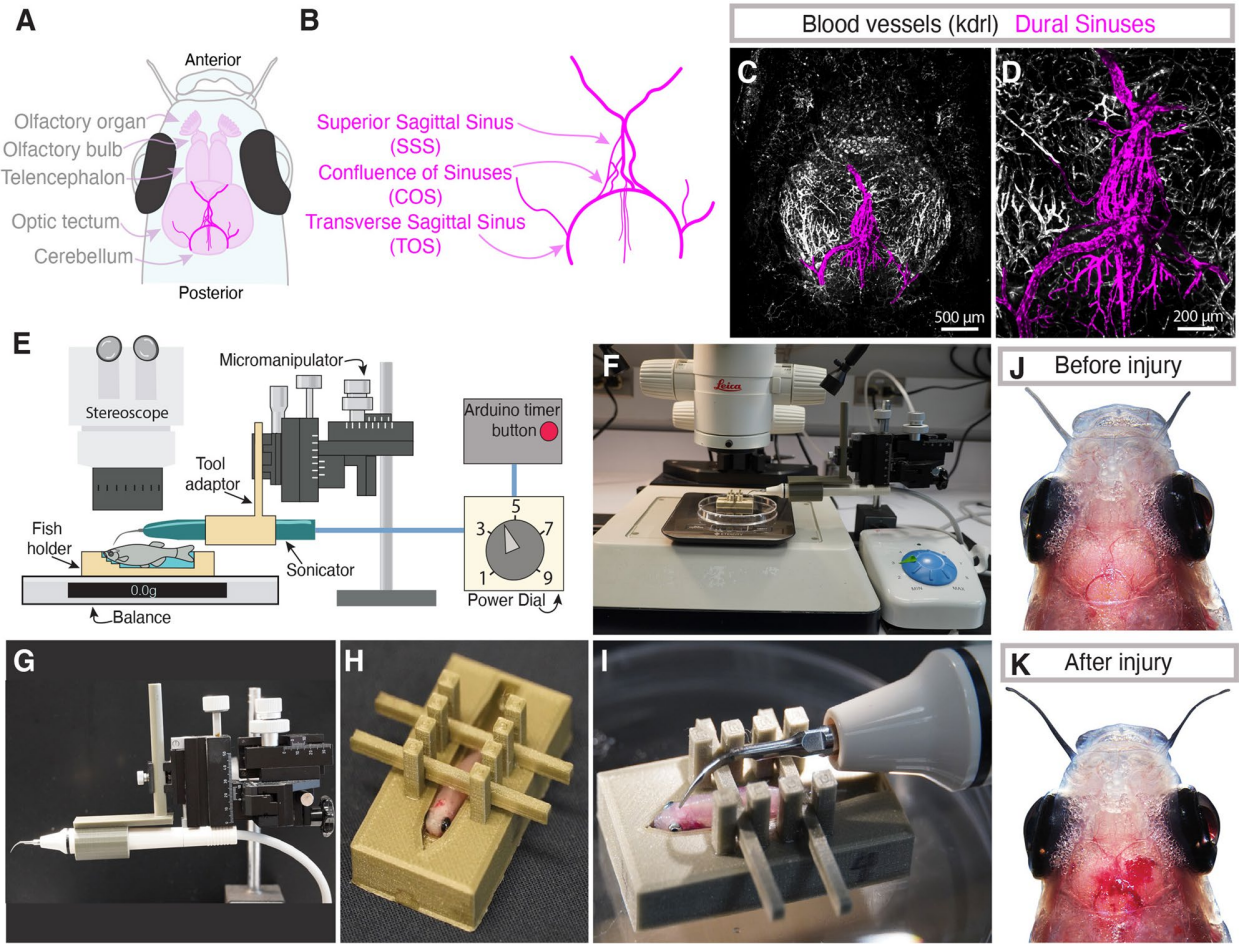
An adult zebrafish model of dural meningeal cerebrovascular injury.** A**,** B**, Schematic diagrams of the dorsal adult zebrafish brain (**A**) and the dural vasculature (**B**), highlighting the presence of the transverse sagittal sinus (TOS) running around the cerebellum, the superior sagittal sinus (SSS) between the two optic tecta, and the confluence of sinuses (COS) where the TOS and SSS meet. **C**,** D**, Confocal images of the dorsal head of a 6-month-old *Tg(kdrl: mcherry)*^*y205*^ animal, with the presumptive dural sinus vasculature pseudocolored in magenta. Panel D shows a magnified view of a portion of the image in panel C. Scale bars are 500 μm (**C**) and 200 μm (**D**). **E**, Schematic diagram of the meningeal cerebrovascular injury (mCVI) apparatus and its composite parts. **F**, Overview image of the mCVI apparatus set up under a stereomicroscope. **G**, Image of the ultrasonic dental scaler attached to a 3D printed adaptor mounted on a micromanipulator. **H**, Image of an adult zebrafish mounted in a 3D printed fish holder with braces to hold the animal in place for the mCVI procedure. **I**, Image of an adult zebrafish with the tip of the dental tool placed positions above the COS for mCVI. **J**,** K**, Images of the dorsal head of a zebrafish before (**J**) and after (**K**) the mCVI procedure, highlighting the highly transparent skull with the brain and major blood vessels clearly visible and the presence of meningeal hemorrhage after mCVI in (**K**). See Supp. Movie 1 for videography of a typical mCVI procedure

In humans, meningeal cerebrovascular injury (mCVI) often involves damage to the dural venous sinuses [17, 27]. Like humans, adult zebrafish have well-developed dural venous sinuses, as readily visualized by confocal imaging of vessels through the translucent skull of *Tg(kdrl: mcherry)*^*y205*^ transgenic (Fujita et al., 2011), melanocyte-free *casper* [57] animals (Fig. 1A-D). The transverse sagittal sinus (TSS) is in the dura above the cerebral ventricles, while the superior sagittal sinus (SSS) runs between the optic tecta, with smaller tributaries above the cerebellum in some individuals (Fig. 1A-B, Supp. Figure 1A-E). The two perpendicular sinuses meet in a plexus of vessels at the confluence of sinuses (COS). While every fish has the same gross anatomy of dural vascular sinuses, similarly to humans the detailed patterning of the vessels varies between individuals (Supp. Figure 1).

We repurposed an ultrasonic dental tool to establish an efficient and inexpensive method to generate localized, reproducible dural mCVI lesions (Fig. 1E-I, Supp. Files 1–12). A custom 3D printed plastic adaptor (Supp. File 12) is used to attach the probe from a Woodpecker UDS-K Ultrasonic Scaler to a stand-mounted micromanipulator for precisely controlled placement of the probe tip (Fig. 1E, G,I). To further facilitate positioning of the probe tip and ensure consistent transduction of dental tool power into head tissue, the adult zebrafish is stabilized by mounting anesthetized animals in custom 3D printed plastic fish holders designed in multiple sizes to accommodate different male and female body forms (Fig. 1H, I, Supp. Files 1–11). The fish holder is placed on a balance scale to measure and control the exact pressure applied by the probe tip on the fish’s head. Three grams of pressure allows for effective lesion generation without breaching the skull at the power levels we employ (see below). A custom-built Arduino controller (Supp. Figure 2) is used to control the length of ultrasonic pulses (programmed for 1 s pulses; Supp. File 14). The entire assembly is placed under a stereomicroscope for magnified observation of the procedure (Fig. 1E, F). This method permits consistent dental tool placement at the same location and rapid generation of reproducible lesions in multiple adult fish (Fig. 1I). Meningeal hemorrhage is clearly visible after inducing mCVI (Fig. 1J-K). The entire procedure (mounting of fish, injury induction, returning of fish to holding tank) can be carried out in approximately 3 min (Supp. Movie 1), and as further discussed below animals given localized, mild mCVI recover from anesthesia and have indistinguishable survival rates from sham treated animals.

### The ultrasonic dental scaler generates scalable meningeal injuries in adult zebrafish

**Fig. 2:**
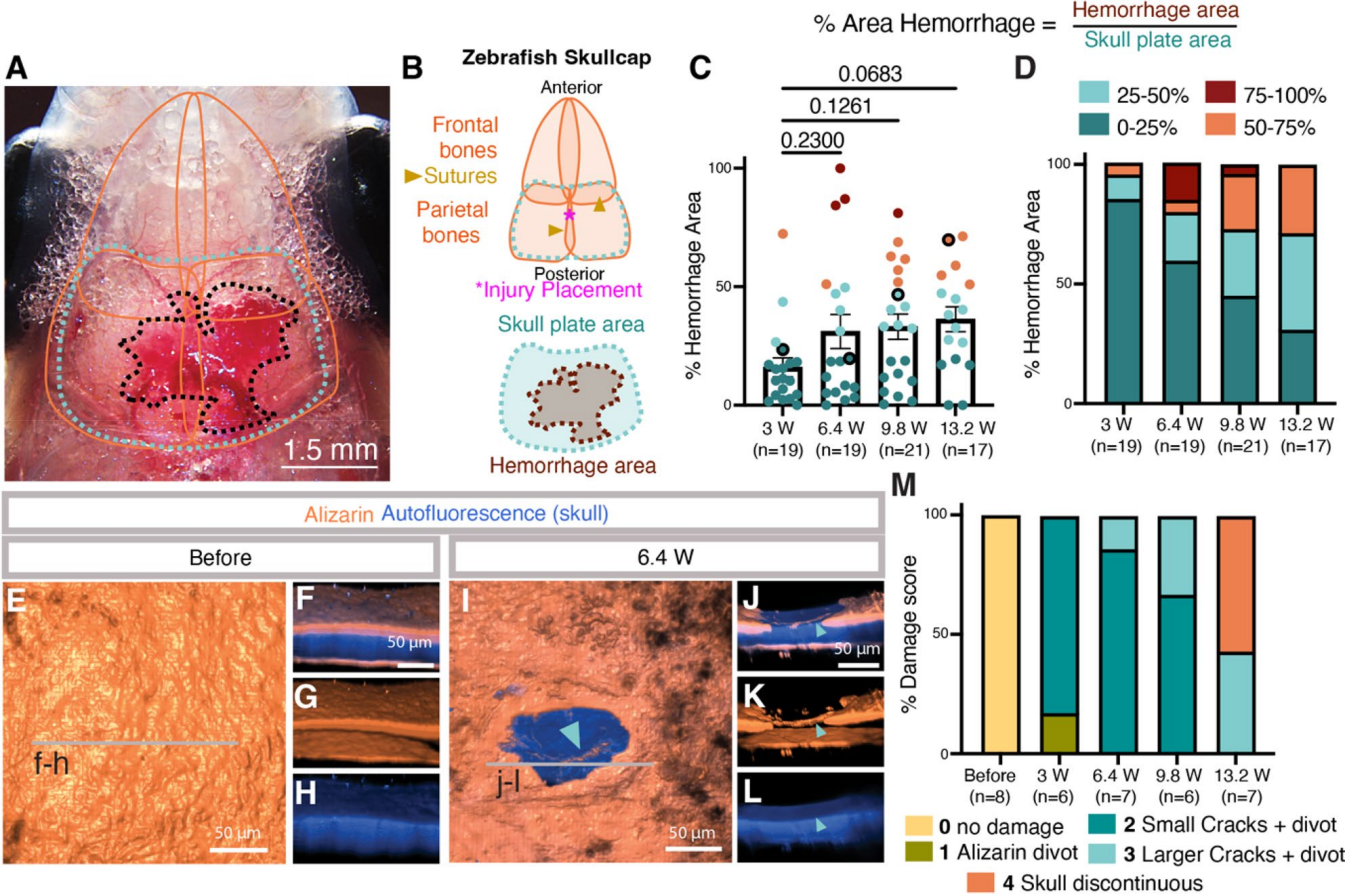
Dural meningeal cerebrovascular injury is scalable and sterile.** A**, Representative image of the dorsal head of an adult zebrafish showing hemorrhage after mCVI (same image as Fig. 1J). Orange lines indicate the perimeters of skull bones underlying the skin; the teal dotted line outlines the parietal skull plate area used to quantify hemorrhage normalized to fish size; the black dotted line marks the hemorrhage area (see panels **B**–**D**). Scale bar, 1.5 mm. **B**, Schematic of parietal and frontal skull bones showing sutures. The dental scaler tip is positioned along the seam between the two parietal bones over the central occipital suture (COS; magenta asterisk). Schematic illustrating the parietal skull plate and hemorrhage areas used to normalize hemorrhage measurement. **C**, Area of hemorrhage for individual fish at four dental tool power settings (3, 6.4, 9.8, or 13.2 W). Bolded points correspond to the photos in Supplemental Fig. 2A-D. One way ANOVA. **D**, Hemorrhage area expressed as a percentage of parietal skull plate area at four dental tool power settings (3, 6.4, 9.8, or 13.2 W). Hemorrhage severity was binned into 0–25%, 25–50%, 50–75%, or 75–100% categories. Numbers of animals analyzed (n) are indicated below the graph. **E-L**, Confocal imaging of alizarin red–stained skull surface bone (orange) and deeper autofluorescent skull bone (blue) on the dorsal skull of wild-type adult zebrafish before (**E–H**) or immediately after (**I–L**) 6.4 W mCVI. Panels show dorsal 3D-rendered surface views (**E**,** I**) or lateral views from the indicated regions, displaying combined signals (**F**,** J**), alizarin alone (**G**,** K**), or autofluorescence alone (**H**,** L**). Scale bars, 50 μm. See Supp. Movie 2 for representative 3D renderings. **M**, Quantification of skull damage following mCVI at four power settings. Scoring criteria are described in Supplementary Fig. 2 and the Methods and Results sections. Numbers of animals analyzed (n) are shown below the graph

We optimized dental tool power with the goal of generating consistent, localized, sterile injuries causing damage to the meninges but not the underlying parenchyma. To quantify hemorrhage, we normalized the visible blood (red) in stereoscope images to overall fish size by calculating the percent hemorrhage area beneath the parietal skull plates (Fig. 2A-B, Supp. Figure 3A-D). The dental tool was positioned above the COS near the intersection of the parietal plates along the skull suture (Fig. 2B). We carried out blinded scoring of hemorrhage at four different tool power (in Watts) levels (3 W, 6.4 W, 9.8 W, or 13.2 W treatments for 1 s each) and found that more power generally increased the likelihood of hemorrhage taking up a greater percent area of the parietal bone area (Fig. 2C-D). We also assessed skull integrity post-injury by using alizarin red (calcium) live staining and the natural autofluorescence of the skull to visualize skull bone (Castranova et al., 2021) at the injury site (Fig. 2E-L, Supp. Figure 3). As we have shown previously (Castranova et al., 2021), brief alizarin red treatment of live animals only stains the outer edges of the skull bone, while the entire thickness of the skull bone is visualized by autofluorescence, allowing for detailed assessment of skull damage and integrity (Fig. 2F). A blinded scoring system was used to evaluate skull damage (Fig. 2M, Supp. Figure 3F-Z). In short, the score is 0 if the alizarin and autofluorescence are entirely intact and there is no damage, 1 when there is a small divot in the outer alizarin stained layer but the skull autofluorescence is intact, 2 when there is an alizarin divot as well as small very superficial cracks evident in the inner blue autofluorescent layer, 3 when cracks are larger, penetrating halfway into the full thickness of the skull but not entirely penetrating the skull, and 4 when the fluorescent signal is discontinuous and the full thickness of the skull is broken (Fig. 2M, Supp. Figure 3F-Z). At 6.4 W there are only minor divots and hairline cracks in the external surface of the alizarin red skull stain, but the internal autofluorescent layers remain intact (Fig. 2I-M, Supp. Movie 2). At 13.2 W cracks penetrate through the skull approximately half of the time (Fig. 2M). Because we wanted a non-penetrative, sterile mCVI model, for all subsequent mCVI procedures described in this study we used 1 s treatment at 6.4 W power with 3 g of pressure to create mCVIs in 8–14 month old adult animals. As noted above, the injury level we employed was specifically calibrated to be survivable and survival was comparable for sham and mCVI treated animals. For the behavior assays described below, 37/42 animals survived sham treatment and 38/42 animals survived mCVI treatment. Failure to survive was due to handling, including injury from netting, fish jumping from dishes to the floor, and failure to revive following intubation with tricaine anesthesia.

### Intracranial damage is largely restricted to the meninges

**Fig. 3:**
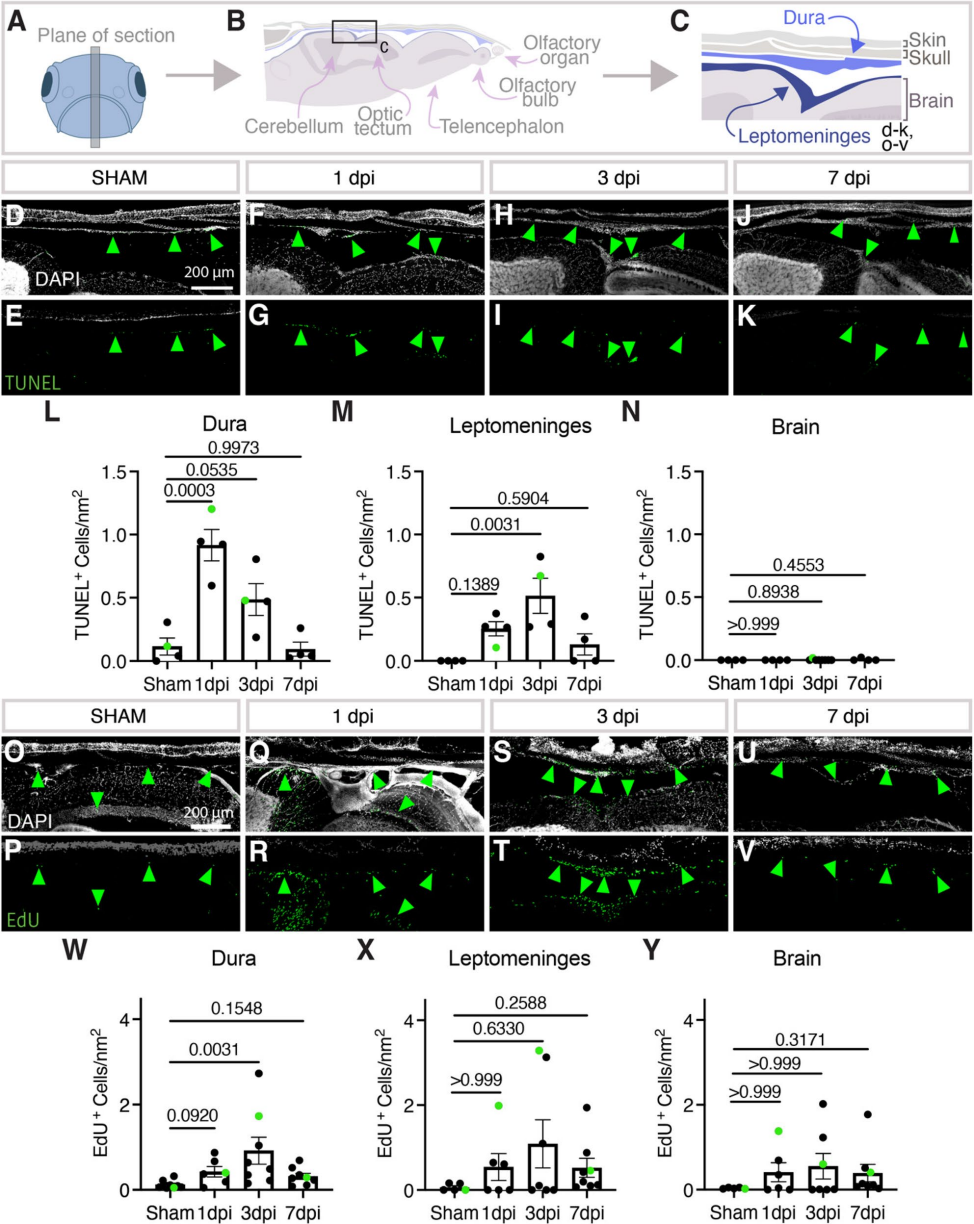
Cell death and cell proliferation after meningeal CVI.** A-C**, Schematic diagrams showing (**A**) the plane of vibratome sectioning used to collect the data in panels D-Y from adult zebrafish, (**B**) depiction and annotation of brain and dorsal skull tissues present in vibratome sections, (**C**) magnified view of the dorsal head from the schematic in panel B with dura and leptomeninges layers highlighted in blue, corresponding roughly to the fields of view in panels **D**-**K** and **O**-**V**. **D-K**, Confocal images of DAPI- and TUNEL-stained vibratome sections prepared as noted in panels **A**-**C**, with DAPI-stained nuclei in white (**D**, **F**,**H**, **J**) and TUNEL staining in green (**D**-**K**). Sections imaged are from sibling adult animals that were either uninjured (**D**, **E**) or 1 (**F**, **G**), 3 (**H**, **I**), or 7 (**J**, **K**) days post-mCVI (dpi). Scale bars are 200 μm. **L-N**, Quantification of the number of TUNEL-positive cells per mm^2^ of section in the dura (**L**), leptomeninges (**M**), and adjacent brain parenchyma (**N**). Statistics are ordinary one-way ANOVA with Dunnett’s multiple comparisons *post hoc* test. **O-V**, Fluorescence microscopy of DAPI- and 5-ethynyl-2′-deoxyuridine (EdU)-stained vibratome sections prepared as noted in panels **A**-**C**, with DAPI-stained nuclei in white (**O**, **Q**,**S**, **U**) and EDU staining in green (**O**-**V**). Sections imaged are from sibling adult animals that were either uninjured (**O**, **P**) or 1 (**Q**, **R**), 3 (**S**, **T**), or 7 (**U**, **V**) days post-mCVI (dpi). Scale bars are 200 μm. **W-Y**, Quantification of the number of EDU-positive cells per mm^2^ of section in the dura (**W**), leptomeninges (**X**), and adjacent brain parenchyma (**Y**). Statistics are non-parametric with Kruskal Wallis with Dunn’s multiple comparisons *post hoc* test

We carried out additional characterization of our model to determine whether damage is localized to the meninges or if the brain parenchyma is also being injured. Vibratome sections were collected from the midline anterior-posterior axis of fixed injured fish heads (Fig. 3A-C). These did not reveal obvious swelling of tissues adjacent to meningeal hematomas, but to assess the level of cell death we used TUNEL staining to measure the number of apoptotic cells in the dura, leptomeninges, and the adjacent cerebellar brain tissue in sham-treated or 1-, 3-, 7-day post injury (dpi) animals (Fig. 3D-N). Interestingly, apoptosis peaks rapidly at 1 dpi in the dura, but peaks at 3 dpi in the leptomeninges (Fig. 3L, M). Importantly, virtually no cell death was observed in the adjacent brain parenchyma (Fig. 3N). We also evaluated cell proliferation following mCVI (Fig. 3O-Y). We carried out intraperitoneal injection of 5-ethynyl-2′-deoxyuridine (EdU) 24 h prior to euthanasia and sacrificed age-matched siblings on the same day to control for baseline proliferation in the adult zebrafish brain. Staining of longitudinal sections reveal that proliferation peaks at 3 dpi in the dura and leptomeninges (Fig. 3W, X). The number of EdU^+^ cells was also increased in the brain parenchyma at 3 dpi, although the identity of the proliferating cells could not be determined (Fig. 3Y). Given that initial cell death is concentrated in the dura, we believe our model primarily damages the dural meninges.

### Aberrant anxiety behavior is associated with mild meningeal cerebrovascular injury

**Fig. 4:**
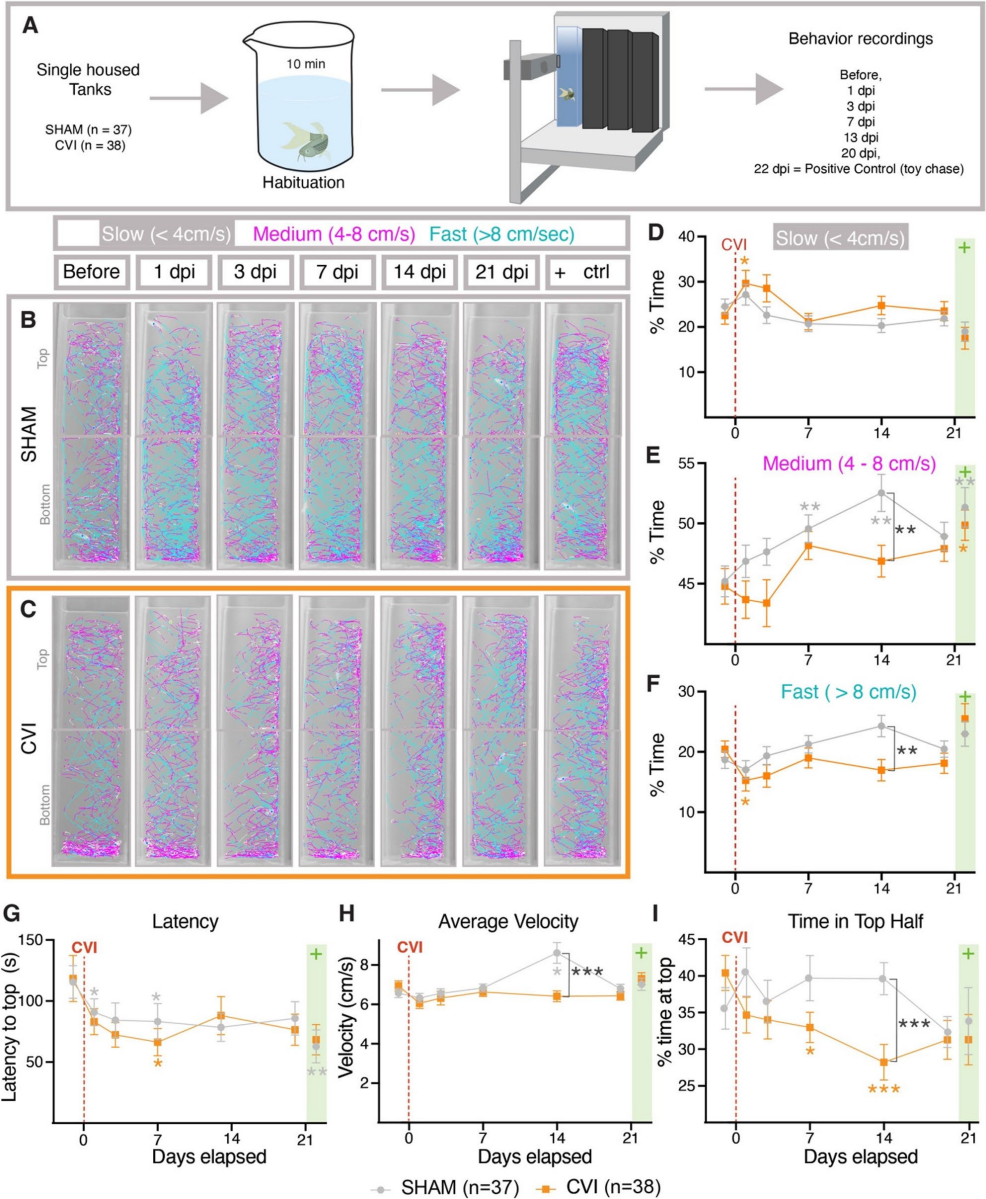
Anxiety responses after meningeal cerebrovascular injury.** A**, Schematic diagram of the diving tank test used to measure anxiety where individually housed animals are habituated in a beaker for 10 min before being introduced to the diving tank and having their movements recorded for 10 min. All animals were measured at initial baseline and at 1, 3, 7, 13, and 20 days after either sham or mCVI treatment. On day 22 post-treatment, fish were chased with a toy predator for 30 s before being netted for habituation and measurement. See Supp. Movie 3 for representative live images of behavior testing. **B**,** C**, Tracks from representative sham (**B**) and mCVI (**C**) treated animals showing the locations of the animals and their approximate speeds (white, < 4 cm/s, pink, 4–8 cm/s, and teal, > 8 cm/s). Both representative animals are male. **D-I**, Quantification of behavioral data for sibling animals at initial baseline and at 1, 3, 7, 13, and 20 days after either sham or mCVI treatment. Graphs show (**D**) percent of time going at slow speed (< 4 cm/s), (**E**) percent of time going at medium speed (4–8 cm/s), (**F**) percent of time going at fast speed (> 8 cm/s), (**G**) latency (time in seconds) to cross to the top half of the tank, (**H**) average velocity (cm/second), (**I**) percent of time spent in the top half of the diving tank. Sham or mCVI treatment on day 0 is noted with a red dashed line. The toy chase on day 22 is noted with green box. For sham group *n* = 37 and fpr mCVI *n* = 38 across 2 independent experimental runs. Statistics are a two-way ANOVA with Dunnett test for multiple comparisons. * *p* < 0.05, ** *p* < 0.01, **** *p* < 0.0001

Mammals exhibit anxiety behaviors after head injuries [58–61]. To assess whether zebrafish subjected to mCVI develop these behaviors, we used the novel diving tank test [52, 62], a commonly used assay for measuring anxiety behavior in adult zebrafish (Fig. 4A, Supp. Movie 3). In this test, more anxious zebrafish should linger in the bottom of the tank longer than less anxious fish. Anxiety read-outs include both latency to enter the top half of the tank (defined as the time from when the fish settles at the bottom to when it first crosses into the top) and percentage of total time spent in the top half of the tank [52, 63]. For each fish, anxiety was measured longitudinally at baseline and at 1, 3, 7, 13, and 20 days post-mCVI or sham treatment. As a positive control for anxiety responses, after completing the 20-day behavioral series following either mCVI or sham treatment, on day 22 fish were chased for 30 s with a 3D-printed plastic toy predator (Supp. File 13), providing an intense, acute net stress–like stimulus [64], before carrying out a final novel diving tank test. Representative swim traces show that mCVI treated animals spent more time moving slowly 1 dpi and lingered at the bottom of the tank especially 7 and 13 dpi, while sham-treated animals maintained consistent movement patterns across longitudinal testing (Fig. 4B-F). For the most part, observed behavioral phenotypes were consistent between sex, though slightly more exaggerated by males (Supp. Figure 4). Latency to the top of tank was slightly faster after mCVI compared to sham-treated animals in the first week, although only significant at 7 dpi (Fig. 4G), and this effect was reduced over subsequent time. Other than on day 13, when a husbandry staff-related incident took place immediately adjacent to the sham-treated animals, mean velocity and therefore distance swum remained relatively constant, suggesting that mCVI did not impair motor control (Fig. 4H). Strikingly, the percent of time spent in the top half of the tank was significantly less for mCVI treated animals compared to sham treated (Fig. 4I). Together these results suggest there is a very small but measurable increase in anxiety-associated behavior after modest dural mCVI.

### Live imaging of the vasculature after meningeal cerebrovascular injury

**Fig. 5:**
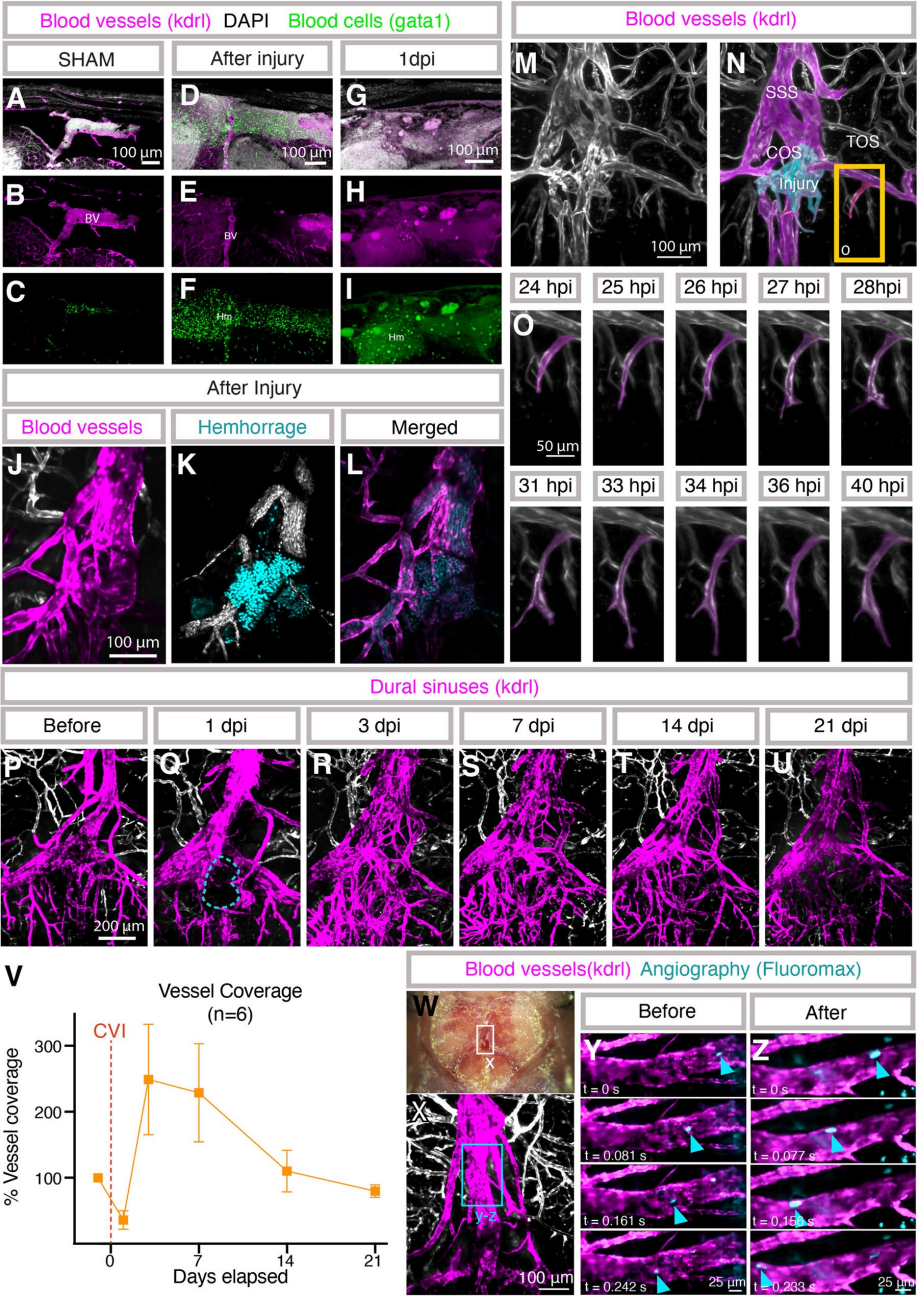
Vascular responses to meningeal cerebrovascular injury.** A-I**, Confocal images of DAPI-stained vibratome sections from adult *Tg(kdrl: egfp)*^*la116*^, *Tg(gata1:dsred*^*sd2*^*)* double transgenic zebrafish prepared as described in Fig. 3**A–C**. DAPI-stained nuclei are shown in white (**A**, **D**, **G**), *kdrl*-positive blood vessels in green (**A**, **B**, **D**, **E**, **G**, **H**), and *gata1*-positive blood cells in magenta (**A**, **C**, **D**, **F**, **G**, **I**). Sections are from sibling animals that were uninjured (**A**–**C**), immediately post-mCVI (**D**–**F**), or 1 day post-mCVI (**G**–**I**). BV, blood vessels; Hm, hemorrhage. Scale bars, 100 μm. **J-L**, Confocal imaging of hemorrhage at the confluence of sinuses (COS) through the intact skull of a living Hoechst-injected *Tg(kdrl: mcherry)*^*y205*^ adult immediately after mCVI. COS vessels are pseudocolored magenta (**J**); circulating blood cells are white and extravasated blood cells teal (**K**); merged view shown in **L**. Scale bars, 100 μm. See Supp. Movie 4. **M-O**, Time-lapse confocal imaging of regrowing dural vessels through the skull of a living *Tg(kdrl: mcherry)*^*y205*^ adult 24 h post-mCVI. M, N show overview images at the start of imaging, with dural vessels in magenta and the injury site in teal (**N**). O shows selected frames of a regrowing vessel within the boxed region in N. Scale bars, 100 μm (**M**, **N**) and 50 μm (**O**). See Supp. Movie 5. P-U, Longitudinal live confocal imaging of dural vessel (*kdrl*-positive) regrowth and remodeling in the same animal immediately before (**P**) and at 1 (**Q**), 3 (**R**), 7 (**S**), 14 (**T**), and 21 (**U**) dpi. **V**, Quantification of vascular coverage in six *Tg(kdrl: mcherry)*^*y205*^ adults, normalized to pre-injury coverage and measured from fluorescence within regions of interest defined at 1 dpi. **W-Z**, Real-time imaging of blood flow through the superior sagittal sinus (SSS) before and immediately after nearby COS mCVI. W shows the imaged region; X shows dural vessels; Y, Z show sequential frames before and after mCVI, with blue arrowheads indicating Fluoromax bead movement. Scale bars, 100 μm (**X**) and 25 μm (**Y**, **Z**). See Supp. Movie 6

Our model and the availability of transgenic reporter lines for endothelial or blood cells permits longitudinal live confocal imaging of dural hemorrhage and dural vascular damage, regrowth, and remodeling in the same animal after mCVI. Imaging of vibratome sectioned *Tg(kdrl: egfp)*^*la116*^, *Tg(gata1:dsred)*^*sd2*^ double transgenic adults confirms extensive hemorrhage after mCVI. Unlike sham-treated animals in which gata1:dsred-positive blood cells are only found within kdrl: egfp-positive blood vessels (Fig. 5A-C), large numbers of extravascular blood cells are clearly visible in the vicinity of the injury immediately after mCVI (Fig. 5D-F). By 1 dpi there are already many fewer intact blood cells in the meninges, although a substantial amount of debris from these cells appears to remain (Fig. 5G-I). Blood cells can also be fluorescently “tagged” and live-imaged concurrently with vessels through the translucent skull by intravenous injection of Hoechst nuclear dye into a *Tg(kdrl: egfp)*^*la116*^ transgenic, *casper* adult animal, confirming extensive extravasation of blood from damaged dural vessels immediately after mCVI (Fig. 5J-L, Supp. Movie 4).

Time-lapse imaging of an intubated [50] *Tg(kdrl: mcherry)*^*y205*^ transgenic *casper* adult animal from 24 to 40 h post-injury (hpi) reveals active angiogenesis of a transverse sagittal sinus (TOS) tributary vessel near the site of injury, in contrast to the quiescent dural vasculature of an uninjured animal (Fig. 5M-O, Supp. Movie 5). Longitudinal assessment of dural vessels can also be carried out over the course of weeks or potentially even months by repeated intubation and imaging of the same animal. We imaged dural blood vessels in a *Tg(kdrl: mcherry)*^*y205*^ transgenic *casper* adult animal just before injury and at 1, 3, 7, 14, and 21 days post-mCVI. The avascular area is revascularized as rapidly as 3 dpi (Fig. 5P-V). By 7dpi, there is an overgrowth of vessels that resolves to close to baseline vascular density by 14 dpi, although some of the vessels are more tortuous compared to the aligned architecture of the dural venous sinus before injury (Fig. 5S, T). By 21 dpi the vascular area has returned to pre-injury vascularity with a similar level of plexus complexity (Fig. 5U-V).

Despite the extensive hemorrhage and evident dural vascular rupture in our model, adult zebrafish all survive injury under our “standard conditions” (1 s treatment at 6.4 W power with 3 g of ultrasonic probe tip pressure), suggesting local blood supply to the dura is largely preserved. To investigate this, we live-imaged blood flow in the superior sagittal sinus (SSS) of an animal immediately (10 min) after mCVI (Fig. 5W-Z; Supp. Movie 6). The SSS is just anterior to the site of mCVI injury at the confluence of sinuses (COS; see Fig. 1A, B for anatomical reference). After intravascular injection of 20 μm FluoroMax beads we see that robust blood flow is still present in the SSS downstream from the mCVI site, suggesting that despite extensive hemorrhage dural vessels are able to maintain or rapidly restore blood supply.

### Live imaging of immune cells after meningeal cerebrovascular injury

**Fig. 6:**
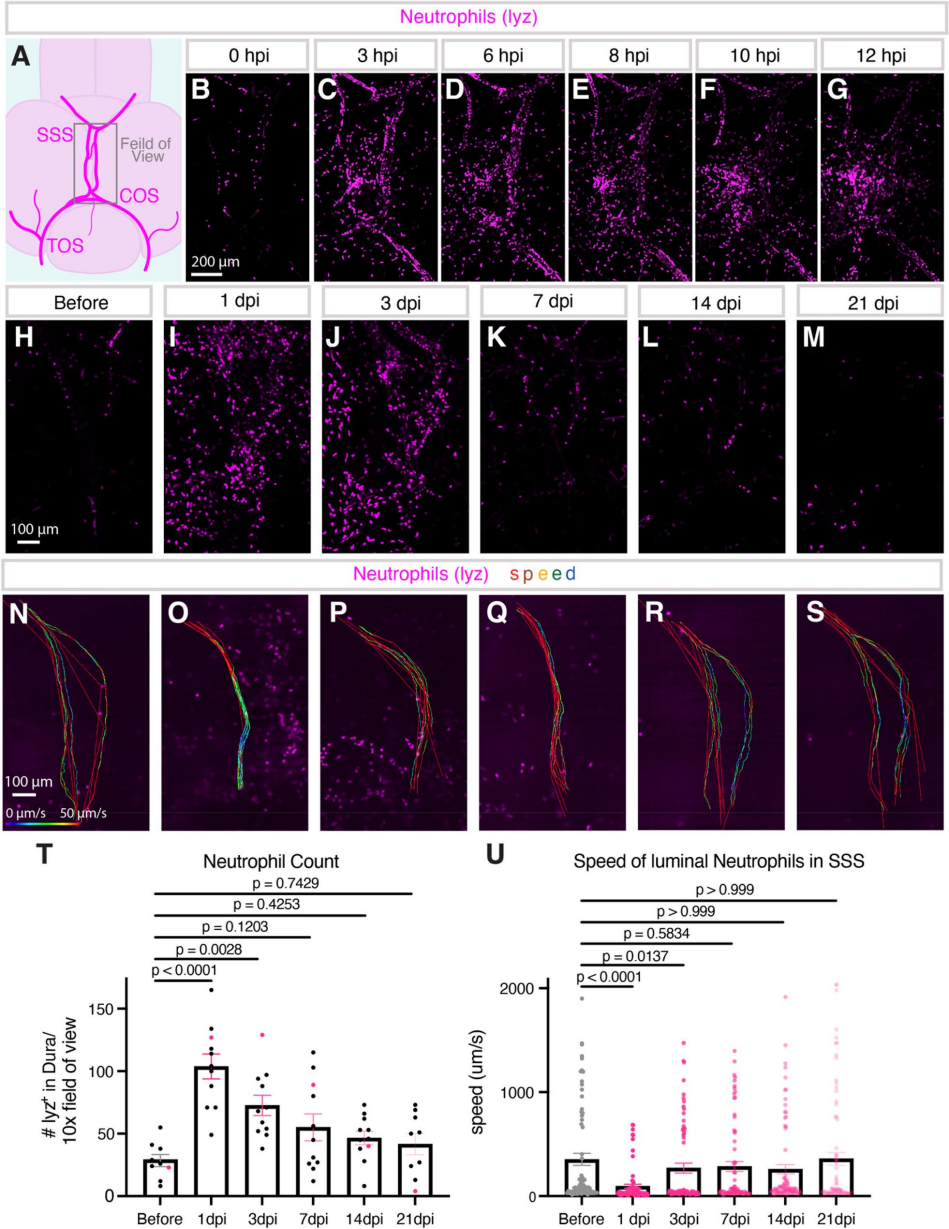
Neutrophils rapidly infiltrate the dura after meningeal CVI and are visualized by live imaging.** A**, Schematic of the anatomy being live imaged in whole intact animals. **B-G**, Stills from Supp. movie 7 showing how increased rolling of neutrophils *Tg(lyz: DsRed2)*^*nz50*^ within hours of injury leads to aggregation of neutrophils in the meninges by 12 h post injury (hpi). **H**, Live imaging of neutrophils in the meninges of zebrafish before injury and longitudinal imaging at **I**, 1 dpi, **J**, 3 dpi, **K**, 7 dpi, **L**, 14dpi, and **M**, 21 dpi of the same animal. Representative tracks of neutrophils moving through the dural superior sagittal sinus (SSS) in the same animal over longitudinal live imaging **N**, before, **O**, 1dpi, **P**, 3 dpi, **Q**, 7 dpi, **R**, 14dpi, and **S**, 21 dpi. **T**, quantification of Neutrophils in the meninges overtime from longitudinal images. *n* = 8 representative of 3 separate experiments. Statistics are one-way ANOVA with Dunnett multiple comparison test. **U**, Speed (µm/s) of neutrophils moving through the SSS. 10 neutrophils per animals were tracked. *n* = 8 representative of 3 separate experiments. Statistics are Kruskal-Wallis with Dunn’s multiple comparison test

After human mCVI, inflammatory mediators such as neutrophil chemoattractants are associated with worse clinical scores after vascular rupture and hemorrhage [25, 65]. Understanding how immune cells such as neutrophils are recruited and how they function at the site of injury to facilitate inflammation is vital but difficult to study in living mammalian models. As a first test to determine whether our model could be used to carry out longitudinal live imaging of immune cell dynamics following mCVI, we imaged neutrophils in *Tg(lyz: dsred2)*^*nz50*^ transgenic [66] animals after dural mCVI. Neutrophils are only sparsely present in the major dural vasculature of uninjured zebrafish, as in mammals [6, 8] (Fig. 6A-B). In a 12-hour time-lapse, z-stacks of the dura were aquired every 10 min (Supp. Movie 7). Within a few hours after mCVI lyz: dsred-positive neutrophils rapidly accumulate around the wounded vasculature (Fig. 6B-G, Supp. Movie 7). By 3 h post-injury (hpi) numerous neutrophils were observed transitint through dural vessel lumens (Fig. 6C, Supp. Movie 7). By 10 hpi neutrophils had densely populated the dural region consistent with local inflammatory recruitment (Fig. 6F). Measurement of neutrophil numbers just before injury and at 1, 3, 7, 14, and 21 days post-mCVI (dpi) shows that neutrophils rapidly peak at 1 dpi, resolving to near-baseline levels by 21 dpi (Fig. 6H-M, T).

The dynamics of individual neutrophils can also be measured longitudinally, including neutrophil transit speed through the injured dural venous sinuses. Live imaging and tracking of neutrophils in the dural venous sinuses of intubated *Tg(lyz: dsred2)*^*nz50*^ transgenic adults just before injury and at 1, 3, 7, 14, and 21 dpi using high-frame rate, single plane imaging reveals a substantial decrease in neutrophil speed through the SSS at 1 dpi compared to pre-injury levels with partial recovery by 3 dpi and a return to baseline by 21 dpi (Fig. 6N-S, U, Supp. Movie 8). Each data point represents the average velocity (µm/s) of a single neutrophil within a single imaging plane (Fig. 6U). A transient decrease in intravascular neutrophil velocity after injury is a well-documented behavior of mammalian neutrophils indicative of increased interactions between blood vessels and leukocytes after inflammation.

## Discussion

Here we present a novel model of meningeal cerebrovascular injury (mCVI) in adult zebrafish generated with an ultrasonic dental scaler. This approach produces focal, sterile vascular injuries in the meninges without breaching the skull or causing cell death in the brain. Although the brain parenchyma remains intact, behavioral assays using the novel tank test revealed a modest increase in anxiety-like behavior after mCVI, suggesting functional consequences of meningeal injury alone. The translucent adult zebrafish skull permits real-time, high-resolution optical imaging of vascular leakage and repair in mammalian-like meninges without invasive surgery. Using transgenic reporter lines and intubation methods, we observed reproducible vascular overgrowth followed by pruning over subsequent days to weeks, as well as rapid neutrophil recruitment through the vasculature. Together, this model provides a tractable and versatile system for dissecting the vascular–immune interactions and inflammatory responses that shape recovery after mCVI. Importantly, unlike established zebrafish brain injury paradigms that primarily target the parenchyma, this model isolates meningeal vascular injury, enabling comparison with and extension of prior work on neuroimmune and regenerative responses in zebrafish.

Adult zebrafish brain injury research has been largely shaped by telencephalic stab lesion paradigms, where ventricular radial glia rapidly exit quiescence, proliferate, and generate new neurons that integrate into injured circuits without permanent glial scarring [56, 67, 68]. Subsequent mechanistic studies demonstrated that acute inflammation is not merely a byproduct of injury, but a trigger of regeneration required for proliferation and neurogenesis [69–72]. More recently, non-penetrating adult TBI models, including weight-drop and focused ultrasound approaches, have extended the field to diffuse and concussion-like injuries, revealing behavioral deficits, neuroinflammation, and temporally coordinated proliferative responses during recovery [55, 73, 74]. Collectively, adult zebrafish brain injury models have advanced our understanding of rapid, tightly regulated immune–regenerative responses to parenchymal injury in vertebrates, in contrast to humans and mice, which mount predominantly inflammatory and gliotic repair responses with minimal neurogenesis and little meaningful brain regeneration. In contrast, our mCVI model is the first to selectively target meningeal vasculature in adult zebrafish, isolating vascular disruption without overt neuronal loss and enabling direct investigation of how meningeal injury alone orchestrates immune recruitment and downstream repair in the brain.

The meninges are increasingly recognized as active immune–vascular hubs rather than passive brain coverings. In zebrafish, the meninges display mammalian-like anatomy, with a highly vascularized dura attached to the skull and underlying leptomeningeal layers [8] Like mammals, zebrafish possess dural venous sinuses that drain blood from the meninges and superficial brain, and we observed inter-individual variability in this network similar to that seen in humans and mice [9, 11, 12, 75] (Supp. Figure 1). These venous structures are closely associated with immune compartments and have been implicated in disorders including infection and meningiomas [1, 3, 76–78]. The zebrafish meninges also contain a full repertoire of innate and adaptive immune cells, mirroring human and mouse systems [7, 8, 79]. This conservation highlights the zebrafish as a powerful model for studying meningeal vascular and immune dynamics in the context of health and disease.

We sought to establish a reproducible model of mild meningeal cerebrovascular injury (mCVI) with scalable control of injury severity. Using an ultrasonic dental scaler with Arduino-timed power control (Fig. 1), we found that increasing power levels produced progressively larger hemorrhages (Fig. 2D). At lower settings, this approach generated a closed-skull, “sterile” meningeal wound, while higher levels occasionally breached skull (Fig. 2E–M). Given that human brain injuries can range from mild and survivable to severe and fatal [34, 80], we tuned the model to reproducibly induce a mild mCVI in which all animals survived, brain parenchyma remained intact, and behavioral changes were modest (Figs. 3N and 5D–I). Notably, even large hematomas had limited vascular damage, showing rapid sealing of dural sinus injury and maintained blood flow by angiography (Fig. 5J–L, Supp. Movies 4,6). This establishes a tractable and physiologically relevant model for studying vascular–immune interactions following mild mCVI.

Zebrafish are already established as neuroimmune models for brain injury, including stroke, ischemia, and spinal cord trauma, owing to their regenerative capacity and amenability to high-throughput testing [35, 67, 81–89]. Prior zebrafish models of traumatic brain injury, including focused ultrasonic injuries that are technically complex, have been directed at studying the brain parenchyma rather than the meninges [55, 84]. Similarly, most vertebrate brain injury models have emphasized neuronal regeneration or inflammation, with limited focus on the impact of vascular damage [83, 87, 90, 91]. Yet hemorrhage in and around the brain is known to drive inflammation, metabolic stress, neuronal death, and poor outcomes when unresolved [23, 92, 93]. Our model directly addresses this gap by creating a precise meningeal vascular injury that disrupts meningeal vessels without killing underlying neurons (Fig. 3N). Despite the brain remaining intact, we observed proliferative responses in adjacent brain tissue (Fig. 3Y), consistent with the high regenerative potential of zebrafish [67, 69, 81, 94]. Indeed stab wound models in adult zebrafish find proliferation of radial glial cells peaks at 2–3 days after wound [95], consistent with the mild proliferation we observed, although proliferation dynamics are different depending on the region of the brain [67, 96]. Unlike mammals, where neuronal precursor cells proliferate after injury but rarely integrate long-term [97, 98], zebrafish progenitors migrate to injured networks, integrate, and differentiate [67, 81], making them uniquely suited to study how vascular injury and immune responses influence neural repair. While we did note proliferation in the meninges and the adjacent brain tissue (Fig. 3W-Y), further investigation will be needed to understand which specific cell populations are proliferating. Our approach therefore provides the first tractable zebrafish system to specifically model meningeal vascular injury and its associated immune responses.

Because even mild traumatic brain injuries in humans and rodents are associated with anxiety [58–61], we tested whether zebrafish exhibit similar behaviors after mCVI. We used the novel diving tank assay (Supp. Movie 3) [52, 63] with tall, narrow infrared tanks (52 cm × 7 × 10.5 cm) previously shown to increase sensitivity in detecting anxiety-like responses [62]. While reduced light at the bottom introduced a scototaxis variable [99], light–dark transitions are also used as an anxiety measure, suggesting this did not confound interpretation. The total distance swum and average velocity did not differ between sham and CVI fish (Fig. 4H), confirming locomotor function was intact. There was one notable exception at day 13 where sham treated fish had higher average velocity (Fig. 4H) likely due to an unplanned husbandry staff-related disturbance, highlighting the sensitivity of laboratory behavior assays. One day after mCVI, fish spend less time swimming at high speeds and more time swimming at slower speeds needed to turn more frequently, resembling the “meander” behavior described in other mild brain injury models [55] (Fig. 4B–F). Latency to enter the top half of the tank was significantly shorter in mCVI fish at 7 dpi (Fig. 4G). Both sham and mCVI groups showed reduced latency over time, consistent with habituation, but the stronger effect in mCVI fish suggests that mild meningeal injury amplifies behavioral adaptation beyond habituation alone (Fig. 4G). Despite this, mCVI-treated fish spent significantly less time in the top half compared to shams, preferring the dark bottom zone and exhibiting less exploratory behavior (Fig. 4I), a classic indicator of increased anxiety in fish [52, 99]. Thus, although modest, anxiety-like behaviors increased in zebrafish following mCVI. Importantly, these findings show that isolated meningeal vascular injury, even in the absence of overt brain damage, is sufficient to produce measurable behavioral changes. This establishes a functional link between meningeal mCVI and altered behavior, paralleling the anxiety phenotypes observed in humans and rodents after mild TBI, and providing a sensitive readout for the downstream impact of vascular–immune interactions in our model.

Studying vascular–immune interactions after CVI has been technically challenging: larval zebrafish models are confounded by ongoing development and neutrophil migration through interstitial tissues, while live imaging in adult mice requires skull thinning that induces inflammation. Larval zebrafish have been instrumental for defining neutrophil biology in myriad wound models, particularly their rapid migration to wounds, egress, and roles in modulating macrophage phenotypes [48, 49, 70, 100–102]. To overcome the limitations of larval and mammalian systems, we applied adult zebrafish intubation methods that permit non-invasive, longitudinal imaging of the meninges [14, 50, 51]. Immediately after mCVI, we observed neutrophils accumulating in the meninges, traveling through the dural vasculature and presumably migrating into injured tissues (Fig. 6A–F; Supp. Movies 7). Furthermore, because the fish remain unharmed during live imaging, we can perform longitudinal time-lapse imaging to analyze vascular–immune interactions, such as neutrophil movement through the dural sinuses, which was slower on average at 1 dpi which correlated to their highest accumulation in the meninges (Fig. 6O-U; Supp. Movies 8). In larval zebrafish brain and spinal cord injury and tail wound models, neutrophil recruitment typically peaks within hours to days and resolves within 2 days as macrophages accumulate [49, 70, 103]. In adult telencephalic stab injury models, neutrophils are similarly among the earliest responders, followed by macrophage-driven resolution and radial glial proliferation [56, 90]. Future work into dynamics of other immune cells in the meningeal compartment will add to our understanding of immune cell cooperation in the meninges. The kinetics observed here are broadly consistent with these paradigms, but occur within a closed-skull meningeal compartment, allowing separation of vascular injury–driven inflammation from parenchymal necrosis. In mouse models of CVI, infiltrating myeloid cells have also been shown to impact microglial phenotypes, promote angiogenesis, and abrogate neuronal damage in adult animals [42, 104]. The adult zebrafish mCVI model provides a unique opportunity to directly interrogate these processes at single-cell resolution in vivo. Because the skull remains intact, future studies into immune cell recruitment, retention, motility and subsequent interactions with injured vessels can be visualized without the confounding inflammation introduced by craniotomy. Thus, the adult zebrafish mCVI model provides an experimentally tractable system to define causal relationships between immune dynamics and vascular regeneration around the adult brain.

In conclusion, we have established a reliable method to generate focal, sterile mCVI in adult zebrafish using an ultrasonic dental scaler. By leveraging the translucent skull, numerous cell type–specific transgenic reporter lines, and recently developed intubation methods, we show that noninvasive, high-resolution imaging of vascular regrowth, remodeling, and immune cell recruitment can be performed repeatedly in the same animal after mild mCVI. Our approach provides a powerful platform for longitudinal analysis of vascular–immune dynamics after mCVI and will enable new insights into the inflammatory mechanisms that shape recovery.

## Materials and methods

### Animal husbandry and strains

Fish were housed in a large zebrafish-dedicated recirculating aquaculture facility (four separate 22,000-liter systems) in 6-liter and 1.8-liter tanks. Fry were fed rotifers, and adults were fed Gemma Micro 300 (Skretting) once per day. Fish were on a typical 14-hour light, 10-hour dark light cycle. Water quality parameters were routinely measured, and appropriate measures were taken to maintain water quality and temperature (80 °F) stability (water quality data available upon request). All fish for imaging and behavior were in a *Casper* –*roy*, *nacre* double mutant [57] – genetic background to increase clarity for microscopy by eliminating melanocyte and iridophore respectively. The following transgenic fish lines were used for this study: *Tg(kdrl: mcherry)*^*y205*^ [105], *Tg(kdrl: egfp)*^*la116*^ [106], *Tg(lyz: dsred2)*^*nz50*^ [66], *Tg(mpeg1:egfp)*^*gl22*^ [107], *Tg(gata1:dsred)*^*sd2*^ [108]. While not shown in the manuscript some of these animals had either *Tg(lyve1:dsred2)*^*nz101*^ [109], *Tg(mrc1a: egfp*)^*y251*^ [110] in the genetic background. Transgenic zebrafish were generated on myriad wild-type backgrounds but are maintained in house by out-crossing to the EK wild-type zebrafish. This study was performed in an American Association for Accreditation of Laboratory Animal Care (AAALAC)-accredited facility under an active research project overseen by the National Institute of Child Health and Human Development Animal Care and Use Committee (NICHD ACUC), Animal Study Proposal # 21 − 015 and # 25 − 015.

### Ultrasonic scaler injury model

To create meningeal CVI, a Woodpecker UDS-K Ultrasonic Scaler was repurposed. In short, the Woodpecker UDS-K Ultrasonic Scaler’s foot pedal was removed and the wires were instead connected to a custom-designed controller (Fig. 1E) consisting of a breadboard (ElectroCookie Solderable Mini Breadboard PCB) with a relay module (AEDIKO Relay Module DC 12 V), a button (Adafruit Stemma Wired Tacticle Push Button pack 4431), and an Arduino (Arduino UNO R4 Wifi and Starter Kit K000007) (Supp. Figure 2). The Arduino was programmed to activate and relay power to the scaler for 1 s after pressing the button (Supp. File 14). All electronic components were placed inside a repurposed container for their protection. A PD1 scaler tip was used for all injuries, and injuries were performed under a Leica (LEICA DFC7000 T) stereomicroscope for accuracy of placement of the scaler on the zebrafish head.

Designs for 3D-printed fish holders, holding braces, and micromanipulator adaptor are provided (see Supplemental Materials). The printed fish holder was designed with a reservoir to hold tricaine system water (0.04%) to keep the fish anesthetized and gills wet, and with a chin rest to minimize head movement during the mCVI procedure (Supp. Files 1–10). Anchoring rods were printed to secure fish to the platform of the fish holder and prevent sliding during injury (Supp. File 11). The ultrasonic scaler was attached to a printed micromanipulator adaptor for spatial control (Supp. File 12). 3D printed items were designed with SketchUp 3D software (SketchUp for Web, 2025) and printed with polylactic acid on an Original Prusa i3 MK3S+ 3D printer (Prusa Research). Models can also be printed by a commercial supplier using the .stl files that we provide (Supp. Files 1–13).

Adult zebrafish aged 6–8 months were anesthetized in 126 mg/L (1x) Tricaine (Tricaine S, version 121718, Syndel; buffered to pH 7 with 1 M Tris-pH9) until they did not react to a fin pinch. The fish were mounted in the 3D-printed fish holder, secured with 3D-printed anchors, and the reservoir was filled with 1x Tricaine. Once the fish was secured, it was placed on a digital scale (SEAUMOON Digital Kitchen Scale) under the stereoscope. A micromanipulator was used to move the 3D-printed adaptor, align the ultrasonic scaler’s tip, and place 3 g of pressure on the visible junction of the optic tecta and cerebellum directly over the confluence of sinuses. The ultrasonic scaler was activated for 1s with a frequency of 28 kHz ± 3 kHz at a power of 3 W (min dial setting), 6.4 W (3 dial setting), 9.8 W (5 dial setting), or 13.2 W (7 dial setting). For sham injuries, animals were anesthetized and then placed in the fish holder with the dental tool placed on the skull for 3 s at 3 g of pressure, without any sonication. Both mCVI and sham procedures were always conducted between 2 and 4 pm, 6–8 h after the lights come on in our fish facility (our fish facility lights are on from 8 am to 10 pm). All experiments have both sexes represented equally. After injury or sham injury, fish were revived with 80 °F system water and observed for 3 h before being transferred to the zebrafish system. On the rare occasions fish failed to recover buoyancy during the 3-hour recovery period, they were removed from the study. Minimal loss of fish from the study also occurred due to commonly encountered handling problems including injury from netting, fish jumping from dishes to the floor, and failure to revive following intubation with tricaine anesthesia. Overall, loss of fish from the study occurred at comparable rates in the sham and mCVI treatment groups (5/42 and 4/42, respectively, in the behavior studies shown in Fig. 4).

### Longitudinal, live confocal imaging

Adult zebrafish that were longitudinally images we individually housed in 0.8 L tanks (Aquaneering ZT080) with a plant for enrichment. Fish were imaged before CVI, and 1-, 3-, 7-, 14-, and 21-dpi. On days fish were live imaged, food was withheld. Intubation of adult zebrafish and imaging of the meninges was conducted as described previously (Castranova et al., 2022; Castranova et al., 2021; Greenspan et al., 2024; Xu et al., 2015). In short, 126 mg/L Tricaine in system water was placed in a 1 L reservoir with an air stone, and a peristaltic pump (World Precision Instruments Peri-Star Pro PERIPRO − 4 L) circulates the water first through a pulse regulator, then to the fish, and then back to the reservoir. The water is kept at 80 °F by wrapping the silicon tubing (Tygon) around a heat block (SH100 Mini Dry Bath Hot Block). The imaging chambers were designed for either inverted or upright microscopes (details below) and the fish were held in place by sponges (anchored by magnets for the upright scope). For longitudinally imaged animals, imaging was conducted between 4 and 12 h after lights come on in our fish facility (our fish facility lights are on from 8 am to 10 pm). Long time-lapse images were initiated approximately 6–8 h after lights on. Fish were imaged for up to 16 h before being revived with 80 °F system water and observed for 3 h before transferred to the zebrafish system.

### CVI hemorrhage quantification

For quantification of hemorrhage area, fish lacking melanophores *nacre*^*−/−*^, were imaged with Leica (LEICA DFC7000 T) stereomicroscope 1 h after injury to ensure hemorrhage into the meningeal space had finished. Using ImageJ, the skull area region of interest (ROI) was drawn around the parietal bone skull plates, whose edges are clearly visible and iridescent in the light images. Then the hemorrhage ROI was drawn around the red blood visible under the transparent skull. The percent area of the hemorrhage was calculated as (hemorrhage ROI area)/(skull plate ROI area)*100 and plotted as a proportion of skull area that had hemorrhage as either 0–25%, 25–50%, 50–75%, or 75–100%.

### CVI skull stain and quantification

For fluorescence imaging of the zebrafish skull, live animals were placed in system water with 0.01% Alizarin for 15 min as described previously [111]. Fish were then rinsed 4 times in fresh system water for 30 min each for residual stain to leave the skin before being live imaged as described above. Alizarin Red S (Sigma Aldrich A5533) was kept as a stock solution of 0.5% pH 7.0 before dilution into system water. The skull has natural autofluorescence in the 405 nm and can be easily captured by boosting the laser power level according to the objective. On the Nikon Ti2 inverted microscope with Yokogawa CSU- W1 spinning disk confocal, Hamamatsu Orca Flash 4 v3 camera, with 20x water immersion 0.95 NA, the power for 405 nm = 95% and for 561 = 98%. Fish were live imaged with the intubation before and after injury and images were then denoised with NIS Elements AR 5.42.04 software using ‘NIS.ai’ and scored blindly. The scoring rubric (examples in Supp. Figure 2F-Z) is as follows: Score 0 is an undamaged skull with all alizarin fluorescence intact. Score 1 has the alizarin stain scraped off the top of the skull. This is likely partially due to the short time of dosing live fish with the alizarin dye meaning that it did not penetrate the full thickness of the skull, leaving surface impacts very easy to visualize. Score 2 had some small surface level cracks inside of the scratched off alizarin stain that did not extend into the skull where the autofluorescence was intact. Score 3 the cracks became more substantial and in some cases extent outside of the alizarin absent divot. Cracks for score 3 were never full thickness through the skull which was easily visualized with the autofluorescence. Score 4 cracks visible with the alizarin correspond with a full thickness break in the autofluorescence through the skull. For all injuries was there was no visible leakage from inside the skull to outside by observation with a stereoscope.

### Fluorescence microscopy

Animals were euthanized according to the animal protocol via ice bath at 1-, 3-, 7-, 14-, and 21-days after CVI or 1 day after Sham injury (explained above). Fish were decapitated and placed in methanol free 4% paraformaldehyde (Electron Microscopy Solutions CAT #15710) buffered to 7.4 pH with 60mM HEPES overnight (not more than 16 h) at 4 °C on a rocker (about 12 full tilts per minute). After fixation, samples were rinsed twice for 5 min in PBS, followed by 5 days in 10% EDTA (pH 7.4) at room temperature. Samples were then rinsed for 24 h in PBS without magnesium or chloride ions (GibocTM CAT #70011044) and sectioned into (150–300 μm) sagittal slices with a Vibrating-feather blade on a microtome (Leica VT1000 S). Sections were stored in PBS at 4 °C until staining.

For Edu staining, 24 hours before euthanasia 4ul of 10mM EdU was intraperitoneally injected as described previously [112]. Vibratome sections underwent EdU staining following the manufacturer’s instructions (Click-iT EdU; Invitrogen, cat# C10340). In short, tissue sections were permeabilized for 20-minute incubation at room temperature in a permeabilization buffer (1% Bovine Serum Albumin (Thermo Fischer CAT #B14) and 0.5% Triton X-100 (Promega CAT #H5142) in PBS). Samples were washed twice for 5 minutes in 1x PBS with 1% BSA before incubation with the EdU solution at room temperature for 1 hour. Sections were washed twice for 5 minutes in 1X PBS solution with 1% BSA, counterstained with DAPI (1:10,000 (Thermo Fisher CAT #D1306)) and cleared with a clearing solution (1 ml glycerol, 6 ml 2,2’-Thiodiethanol, 3 ml 1X PBS) [113, 114] for 24 h at 4 °C on a slow rocker. Samples were mounted in clearing solution before imaging.

For TUNEL staining, tissue sections were permeabilized with 20 µg/ml proteinase K in PBS for 5 min on a circular rotating table at 50 rpm before 2 × 5-minute washes in PBS. Sections were post-fixed in 4% paraformaldehyde for 15 min and washed 5 times for 5 min with PBST (PBS with 0.01% Triton X-100). Afterwards, samples were further permeabilized with a 10-minute incubation in a permeabilization buffer (0.1% Triton X-100 and 1% sodium citrate (Thermo Fischer CAT #J63888.AK) in PBS). Sections were washed twice for 5 min with PBST and stained with TUNEL using kit instructions (TUNEL Assay Apoptosis Detection Kit, Biotium CAT #30074). Samples were incubated with TUNEL Reaction mix, which included 2.5 µL of TdT Enzyme to 50µL of TUNEL Reaction Buffer and DAPI for one hour at 37 °C on a rocker in the dark. Sections were washed twice with PBST for 5 min before DAPI counterstain and cleared with LUCID.

Confocal image stacks were collected from the thick (150–300 μm) sagittal vibratome sections of the head on a Nikon FNSP upright microscope with AXR scanning confocal. Images were acquired with a 20x water immersion objective (1.0 NA) with sequential resonance bidirectional mode with 8x averaging per channel, 1024 × 1024 pixel ratio, and 1.14 zoom using the Nikon Elements Advanced Research software. 405 nm, 488 nm, 561 nm, and 640 nm laser lines were used with an emission range of 429–474 nm, 499–551 nm, 571–625 nm, and 662–737 nm, respectively. Z-series stacks were captured with a 1.5-µm step size and were processed, denoised, and stitched with Nikon Elements. Maximum Intensity Projected images of 21 optical sections from the same anatomical depth within the 150–300 μm sagittal vibratome sections of the head were imported to ImageJ for cell count image analysis. To quantify EdU^+^ or TUNEL^+^ cells, regions of interest were drawn around the dura, choroid plexus, leptomeninges, and superior cerebellum adjacent to injury using the Fiji polygon tool. Fiji Auto Threshold v1.18.0 Shanbag or Yen methods for Edu and TUNEL respectively. Cells were quantified using the Analyze Particles feature in Fiji with a set size of 3µm^2^-Infinity for EdU^+^ cells and 1µm^2^-Infinity for TUNEL^+^ cells.

### Behavior

Custom-designed infrared (IR) permeable-plastic tanks (Precision Plastics Inc.) were used for the diving tank assay as previously described [62]. These tall tanks (7 cm width, 52 cm height, 10.5 cm length) were designed with Infrared Transmitting Acrylic, making them impervious to visible light but not IR. Using the ZebraTower platform (Viewpoint Behavior Technology), 4 tanks faced the IR camera (maximum 30fps) with live tracking 2D sideview ZebraLab software (Viewpoint Behavioral Technology) to track and record locomotion. When running the assay CVI and SHAM treatments were every other animal to account for slight bench effects between runs as 2 animals were always CVI and 2 were SHAM for each run. 10-minute recordings were integrated into 5-minute periods. ‘Background refresh delay’ was set to 16 s, ‘detection sensitivity’ was set to 10, movement thresholds were set to 8 for ‘large’ and 4 for ‘inactivity’, and ‘X minimum size’ was set to 15. Each tank had 2 ROIs – one on the top half and one on the bottom. Adult Zebrafish were housed separate in 1.8 L tanks at least 24 h before diving tank assay. Before each experiment, fish were netted into a 600mL beaker with 200mL system water and kept in darkness for 10 min to avoid aversion behavior from netting and seeing researchers. Fish were then carefully poured into the behavior area for a total volume of 2 L of system water. After recording, each fish was transferred into their individual home tanks, and the IR tanks were cleaned with 70% ethanol and rinsed with system water between each run. Every fish was assayed once per time point, for a total of 6 assays per fish. All experiments were performed between 1:00 pm and 6:00 pm, or 5–10 h into daylight cycle. As a positive control for anxiety response, a PLA-printed predatory toy (Supp. File 13) attached to a stick and approximately 20x the volume of a zebrafish was used to chase the fish around their home tanks for 30 s before being put in the beaker for the habituation period and undergoing the novel tank test. The 30 s chase period was timed, and the researchers attempted to follow the fish without damaging collisions at a speed that did not spill water from the 1.8-liter Aquaneering tank it was housed within as would be done in a net chasing protocol. This was done to simulate a net-chasing assay to induce anxiety [64].

For analysis of diving tank assay, the last 5 min integration period of the recording was used for % Time going slow, medium, or fast, the distance and velocity calculations, and % time in the top half of the diving tanks. Latency as the time it took the fish to enter the top half of the tank, was calculated by a researcher blindly recording the time after being transferred into the diving tank the fish settled at the floor of the tank and the time the animal crossed into the top ROI then calculating the seconds in between. As indicated above, slow speed was when the fish moved less than 4 cm/s, medium speed was between 4 and 8 cm/s, and fast was any speed greater than 8 cm/s. The tracks shown are also from the second 5-minute integration period as the calculations.

### 3D-cell live cell counts

Neutrophils were counted automatically using the Spots tool in IMARIS software. The cell diameter was set to 15 μm for the XY plane and 30 μm for the Z plane to account for Z-stretch during imaging and cell movement. Cells were tracked using the far-red channel with background subtraction enabled and counted within a restricted 3D region of interest (650 μm Width, 650 μm length, 110 μm depth) around the area of injury. Neutrophil spots were manually removed if incorrectly assigned to debris or autofluorescence.

### Neutrophil tracking

Imaris Cell Tracking Software (Version 9.9.0) was used for semiautomated immune cell tracking. Confocal microscopy was used to generate fluorescent z-stack 10-minute movies which then were converted to 2-dimensional maximum intensity projections in Imaris. The “spots” tool was used for manual tracking of cells, with the “auto-connect to selected spot” setting enabled to facilitate continuous tracking. The total number of frames was divided by 10, and the movie was examined at each frame point, equally spaced, for a spot to follow from one side of the vessel to the other. Spots were manually tracked by every frame that they were seen. Computation of track speed and displacement was done by the Imaris distance-detection Matlab algorithms within the software. The color mapping of speed on Imaris tracks was done in the “color” tab, setting the color type to “statistics coded” for speed, and applying it to the tracks. Every image kept the colormap on the “spectrum” setting with a range of 0-150 for visualization of track speed.

### Quantification for vessel regrowth

To quantify vessel regrowth, a region of interest was made around the avascular area at every time point using the polygon tool in Fiji. The area of the region of interest was measured, and the percentage of vessel regrowth was calculated as described previously [51].

### Statistics

All statistics were done in Graphpad prism 10.0.2 (171) as described in the figure legends. * *p* < 0.05, ** *p* < 0.01, **** *p* < 0.0001.

### Arduino resources

Breadboard circuit- an image with legend showing how everything is connected is in Supplemental Fig. 2 and Kraus et al. Supp, File 14.

## Supplementary Information

Supplementary Material 1.

## Data Availability

All data are available upon request.

## Abbreviations

TBI: Traumatic brain injury
mCVI: Meningeal cerebrovascular injury
BBB: Blood brain barrier
TSS: Transverse sagittal sinus
SSS: Superior sagittal sinus
COS: Confluence of sinuses
Dpi: Days post injury
BV: Blood vessels
Hm: Hemorrhage
