# Supplementary material for: Longitudinal in vivo imaging of dynamic immune–vascular responses to meningeal cerebrovascular injury
Source: J Neuroinflammation. 2026 Mar 17;23:141. doi: 10.1186/s12974-026-03767-6 (PMC13107746; doi:10.1186/s12974-026-03767-6)

**SUPPLEMENTAL FIGURE LEGENDS**

**Supplemental Figure 1. Heterogeneity of the dural vasculature.** **A,** Pseudocolored confocal image of the dorsal head of a 6-month-old *Tg(kdrl:mcherry)^y205^* animal (same image as in Fig. 1C), annotated with the dural sinus vasculature pseudocolored in magenta and labeled, and the locations of other vessels in the image noted with other colors. **B-E,** Confocal images of the dorsal head of a 6-month-old *Tg(kdrl:mcherry)^y205^* shown at different magnifications, with the dural sinus vasculature pseudocolored in magenta in panel E. **F-S,** Confocal images of the dorsal heads of fourteen different 6-month-old *Tg(kdrl:mcherry)^y205^* siblings, revealing variation in the detailed pattern of the dural sinus vasculature (pseudocolored in magenta), despite similarity in their gross patterning. The bottom half of each panel shows a magnified portion of the image in the top half of the panel. Scale bars = 500 μm (A), 500 μm (B), 200 μm (C), 200 μm (D,E), 500 μm (top halves of F-S), 200 μm (bottom halves of F-S).

**Supplemental Figure 2. Circuit diagram for digital control of ultrasonic dental scaler.** This circuit replaces the foot pedal of an ultrasonic dental scaler, enabling precise, millisecond-level digital control of activation timing for use in a zebrafish injury model. The circuit consists of three components: an Arduino Uno microcontroller (A), a push-button module (B), and a 5V relay module (C). When the button (B) is pressed, it sends a 5V HIGH signal to the Arduino’s (A) digital pin D2, allowing portable, standalone operation without a connected computer. The Arduino (A) then sends a 5V HIGH signal from digital pin D9 to activate the relay (C), which closes its normally open (NO) contacts and completes the circuit that powers the dental scaler. The relay (C) provides electrical isolation, enabling the low-voltage Arduino (A) to safely control the higher-voltage AC circuit used by the scaler.

**Supplemental Figure 3. Hemorrhage and effects on the skull after dural meningeal cerebrovascular injury. A-D,** Representative images of dural hemorrhage at 3 W (A), 6.4 W (B), 9.8 W (C), and 13.2 W (D) of power. Scale bars are 1.5mm. **E,** Schematic diagram of experimental workflow for Alizarin red staining, mCVI, and skull imaging. See Methods section for additional details. **F-Y,** Representative confocal images of alizarin red-stained skull surface bone (orange) and deeper autofluorescent skull bone (blue) on the dorsal skull of wild type adult zebrafish siblings before that were uninjured (F-I) or subjected to mCVI with 3 W (J-M), 6.4 W (N-Q), 9.8 W (R-U), or 13.2 W (V-Y) of power for 1 second. Images shown in panels are dorsal views 3D rendered to show “solid” views of the skull surface (F,J,N,R,V) or lateral views of a portion of the same confocal image stacks beginning from the lines on panels E and H, with alizarin + autofluorescence (G,K,O,S,W), alizarin alone (H,L,P,T,X), or autofluorescence alone (I,M,Q,U,Y). Scale bars = 50 µm. **Z,** Scoring rubric used to determine values graphed in Fig. 2M.

**Supplemental Figure 4. Anxiety responses after meningeal cerebrovascular injury by sex. A-L,** Quantification of behavioral data for sibling animals at initial baseline and at 1, 3, 7, 13, and 20 days after either sham or mCVI treatment. Graphs show latency (time in seconds) to cross to the top half of the tank over time is graphed for (A) Males, and (B) Females, average velocity (cm/second), for (C) Males and (D) Females, percent of time spent in the top half of the diving tank for (E) Males and (F) Females, and percent of time going at slow speed (<4 cm/s) for (G) Males and (H) Females, percent of time going at medium speed (4-8 cm/s) for (I) Males and (J) Females, percent of time going at fast speed (>8 cm/s) for (K) Males and (L) Females. Sham or mCVI treatment on day 0 is noted with a red dashed line. The toy chase on day 22 is noted with green box. For sham group n = 37 and for mCVI n = 38 across 2 independent experimental runs. Statistics on the effect of time within each treatment are a two-way ANOVA with Dunnett test for multiple comparisons. Statistics on the effect of treatment on within each time point are a two-way ANOVA with a Tukey’s test for multiple comparison. * p < 0.05, ** p < 0.01, *** p < 0.001.

**SUPPLEMENTAL MOVIE LEGENDS**

**Supplemental Movie 1. Method of meningeal cerebrovascular injury.**  A movie showing (1) an overview of the apparatus used to induce meningeal cerebrovascular injury (mCVI) in adult zebrafish, (2) higher magnification view of a hemorrhaging mCVI lesion being generated in the dural meninges of an adult fish, and (3) an adult fish swimming after mCVI injury.

**Supplemental Movie 2. Zebrafish skull remains intact after mCVI.** Overview schematic diagram and confocal images of alizarin-stained live adult zebrafish before and after mCVI at 6.4 W power. Alizarin labeling of the superficial skull (orange) and autofluorescence of the deeper skull (blue) shows that the skull remains intact after mCVI.

**Supplemental Movie 3. Novel tank diving test assay after mCVI.** Representative example video of a novel tank diving test to measure adult zebrafish anxiety. Tall test tanks (7 cm width, 52 cm height, 10.5 cm depth) opaque to visible light but transparent to infrared light were recorded with an infrared camera for 10 min (only 1-1/2 minutes shown). Fish occupancy of either the bottom or top of the tank was assessed in addition to other parameters. The time from when the fish settles on the bottom of the tank to when it first transitions to the top of the tank was recorded as the latency, an established measurement of anxiety. In the example shown here fish were tested at 3 days post-sham or mCVI injury,

**Supplemental Movie 4. Hematoma forms around dural venous sinuses after mCVI.** Transcranial confocal imaging of red blood cells stained with Hoechst nuclear dye (teal) flowing through the dural venous sinuses (magenta) of a *Tg(kdrl:mcherry)^y205^* transgenic adult zebrafish before (left) and after (right) mCVI. Hematoma is visible as stationary blood cells.

**Supplemental Movie 5. Vascular plasticity after mCVI.**  Ten hour long live time-lapse transcranial confocal imaging of the dural venous sinuses (white) in uninjured (left) and 1 day post-mCVI (right) intubated adult *Tg(kdrl:mcherry)^y205^* transgenic adult zebrafish. Vessels are quiescent in the uninjured animal, but active angiogenesis is taking place in the vicinity of the injury in the animal with mCVI (example highlighted in teal).

**Supplemental Movie 6. Dural superior sagittal sinus blood flow is retained after mCVI.** Live transcranial confocal imaging of blood flow through the superior sagittal sinus (SSS) before (top) and immediately after (bottom) induction of mCVI at the nearby confluence of sinuses (COS) in an adult *Tg(kdrl:mcherry)^y205^* transgenic adult zebrafish (magenta) intravascularly injected with fluoromax beads (teal), showing that robust blood flow is maintainined through the SSS after COS mCVI.

**Supplemental Movie 7. Neutrophil dynamics in the injured dura immediately after mCVI.** Twelve hour live transcranial confocal time-lapse imaging of neutrophils (magenta) in the dural plane of an intubated adult *Tg(lyz:dsred2)^nz50^* transgenic animal, beginning immediately after mCVI injury. One z-stack through the dura every 10 min. Neutrophils are initially sparsely distributed in the meninges but accumulate rapidly after injury, first rolling through the nearby dural sinuses and then accumulating in the meninges around the site of injury.

**Supplemental Movie 8. Neutrophils move slower through the dural sinuses after injury.** Ten-minute-long time lapse (only 6-9 minuts shown) of a single plane (3.3 frames per second) through the dura of an intubated adult *Tg(lyz:dsred2)^nz50^* transgenic fish before and 1 day after mCVI. Commit tails show relative speed of neutrophils (Magenta) travelling through the superior sagittal sinus with red being fast and blue being slower. Magenta arrows highlight neutrophils moving through the vasculature lumen. Neutrophils in the meningeal tissue were not tracked for vascular interactions for this study.

**SUPPLEMENTAL FILES**

**Supplemental File 1** CAD design for 3-D printed platform used to restrain adult fish for the mCVI procedure, designed with posts for securing bracing pins (Supp. File 12) and with a water reservoir for keeping the fish moist. Cavity width is 6mm at the top and 4mm at the base with a depth of 6mm.

**Supplemental File 2** CAD design for 3-D printed platform used to restrain adult fish for the mCVI procedure, designed with posts for securing bracing pins (Supp. File 12) and with a water reservoir for keeping the fish moist. Cavity width is 6mm at the top and 4mm at the base with a depth of 4mm.

**Supplemental File 3** CAD design for 3-D printed platform used to restrain adult fish for the mCVI procedure, designed with posts for securing bracing pins (Supp. File 12) and with a water reservoir for keeping the fish moist. Cavity width is 6mm at the top and 3mm at the base with a depth of 4mm.

**Supplemental File 4** CAD design for 3-D printed platform used to restrain adult fish for the mCVI procedure, designed with posts for securing bracing pins (Supp. File 12) and with a water reservoir for keeping the fish moist. Cavity width is 6mm at the top and 3mm at the base with a depth of 5mm.

**Supplemental File 5** CAD design for 3-D printed platform used to restrain adult fish for the mCVI procedure, designed with posts for securing bracing pins (Supp. File 12) and with a water reservoir for keeping the fish moist. Cavity width is 4.3mm at the top and 3.1mm at the base with a depth of 4.4mm.

**Supplemental File 6** CAD design for 3-D printed platform used to restrain adult fish for the mCVI procedure, designed with posts for securing bracing pins (Supp. File 12) and with a water reservoir for keeping the fish moist. Cavity width is 4.4mm at the top and 3.1mm at the base with a depth of 3.5mm.

**Supplemental File 7** CAD design for 3-D printed platform used to restrain adult fish for the mCVI procedure, designed with posts for securing bracing pins (Supp. File 12) and with a water reservoir for keeping the fish moist. Cavity width is 6mm at the top and 4mm at the base with a depth of 6.8mm.

**Supplemental File 8** CAD design for 3-D printed platform used to restrain adult fish for the mCVI procedure, designed with posts for securing bracing pins (Supp. File 12) and with a water reservoir for keeping the fish moist. Cavity width is 6mm at the top and 4.5mm at the base with a depth of 6.5mm.

**Supplemental File 9** CAD design for 3-D printed platform used to restrain adult fish for the mCVI procedure, designed with posts for securing bracing pins (Supp. File 12) and with a water reservoir for keeping the fish moist. Cavity width is 6mm at the top and 5mm at the base with a depth of 7mm.

**Supplemental File 10** CAD design for 3-D printed platform used to restrain adult fish for the mCVI procedure, designed with posts for securing bracing pins (Supp. File 12) and with a water reservoir for keeping the fish moist. Cavity width is 6mm at the top and 4.5mm at the base with a depth of 4.5mm.

**Supplemental File 11** CAD design for 3-D printed bracing pins used to secure adult fish onto the fish bed (Supp. Files 1-10) during injury and prevent movement while the fish is under anesthesia.

**Supplemental File 12** CAD design for 3-D printed adapter designed to hold the ultrasonic dental scaler during the mCVI procedure and mount the appliance onto a micromanipulator for precise control.

**Supplemental File 13** CAD design for 3-D printed novel object with predatory features at 20x the size of an adult zebrafish, used to chase fish for 30 seconds to induce anxiety as a positive control for the novel diving tank test.

**Supplemental File 14**  Code used to program an Arduino UNO R4 Wifi for one-second activation of a Woodpecker UDS-K Ultrasonic Scaler.

**Supplemental File 15** Behavior data as output from Viewpoint software recorded over 5 min and Latency calculated as difference between when fish reached the bottom of the tank and the time at which it entered the top of tank ROI.


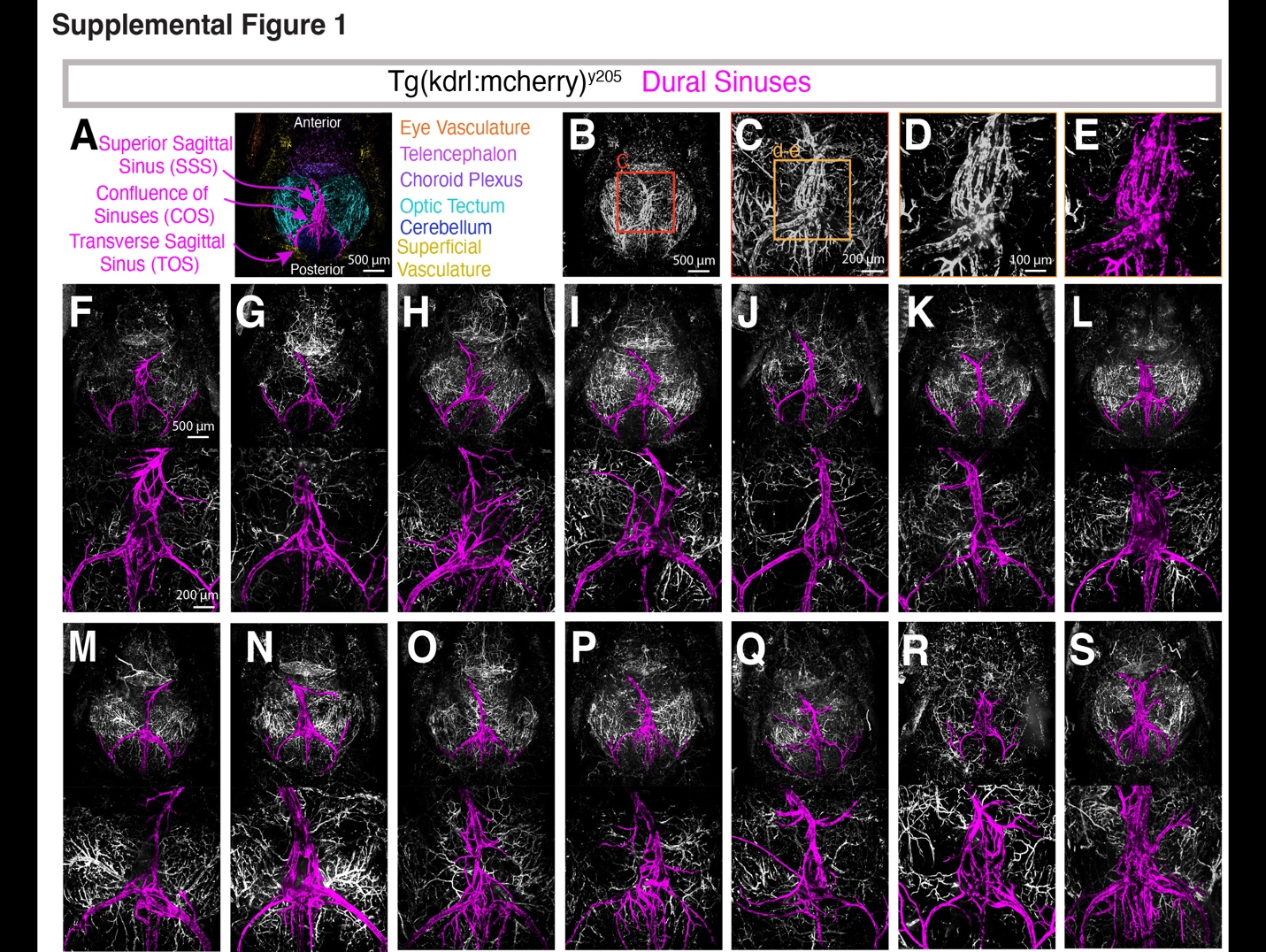

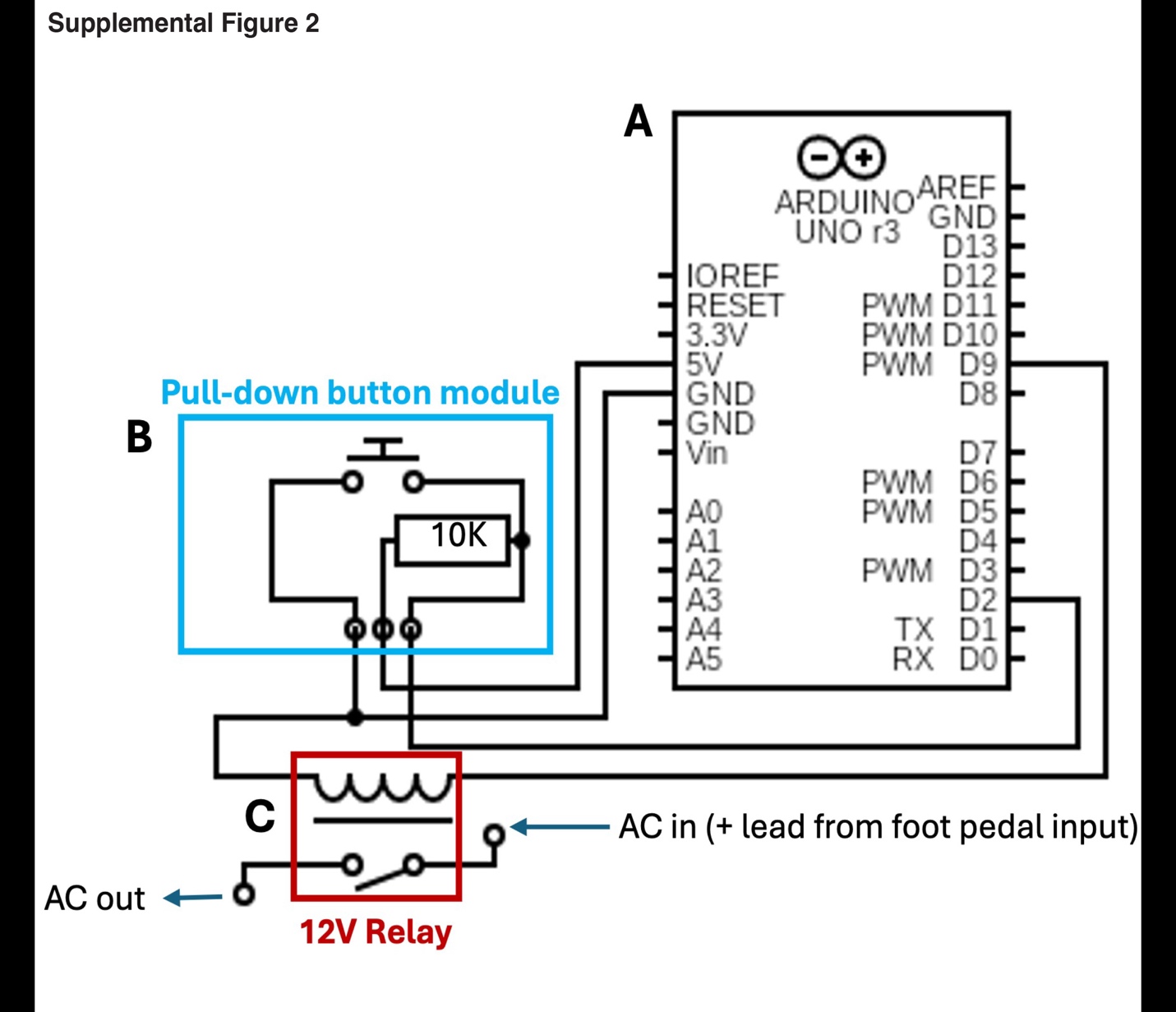

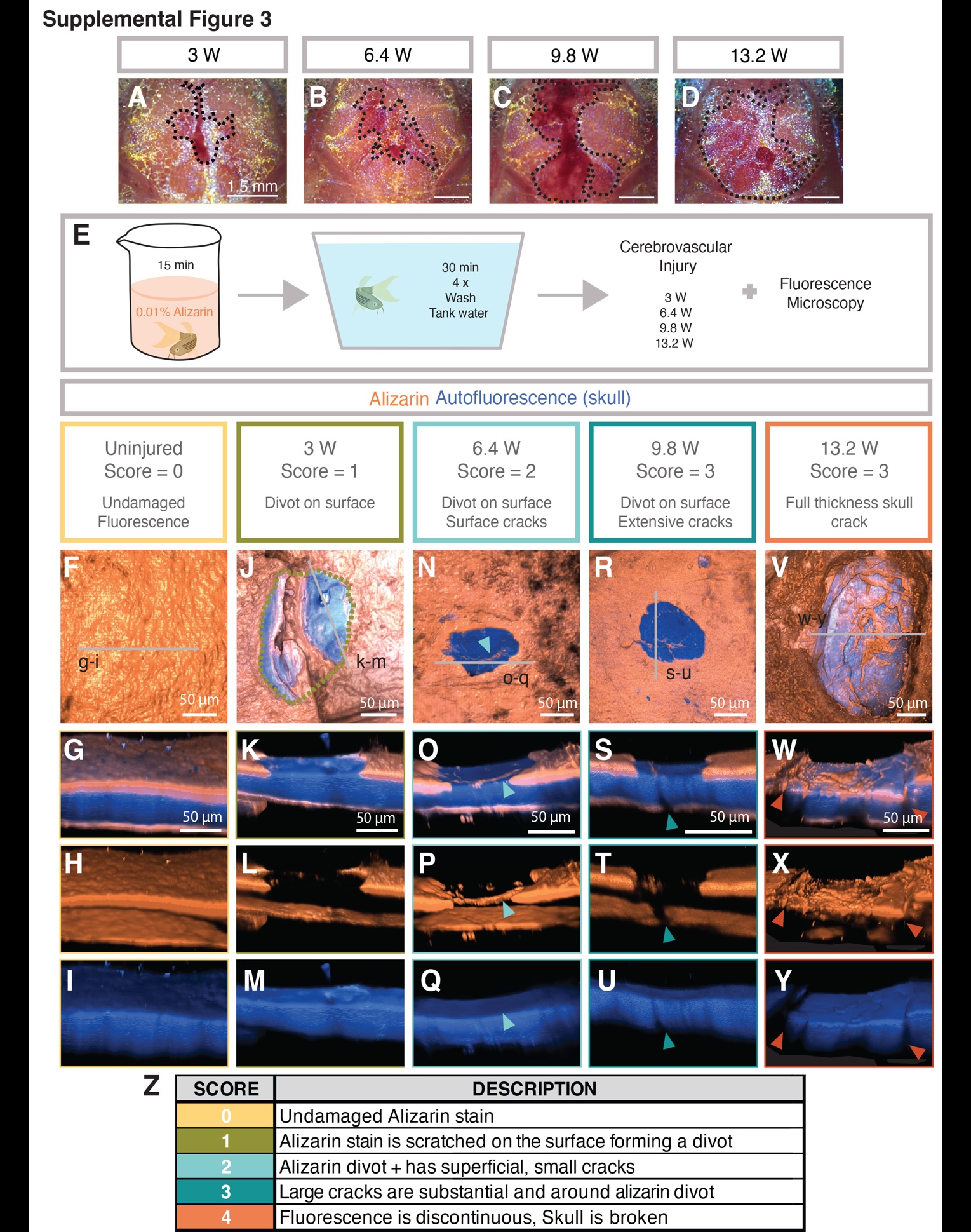


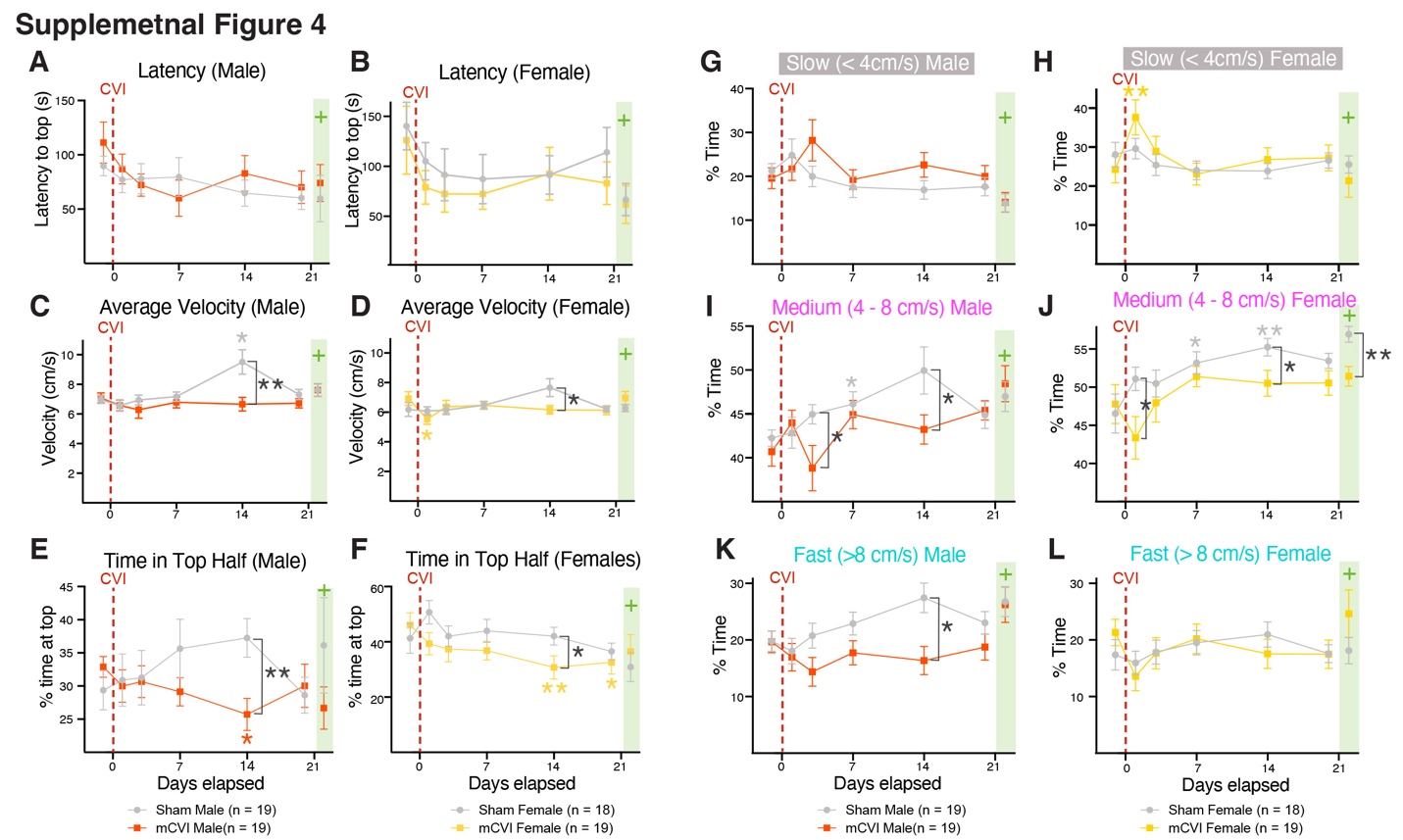

Supplement: Supplementary file 1 — Supplementary Material 1. [file 12974_2026_3767_MOESM1_ESM.docx]
